# Recruitment of monocytes primed to express heme oxygenase-1 ameliorates pathological lung inflammation in cystic fibrosis

**DOI:** 10.1038/s12276-022-00770-8

**Published:** 2022-05-17

**Authors:** Caterina Di Pietro, Hasan H. Öz, Ping-xia Zhang, Ee-chun Cheng, Valentino Martis, Tracey L. Bonfield, Thomas J. Kelley, Ronald Jubin, Abraham Abuchowski, Diane S. Krause, Marie E. Egan, Thomas S. Murray, Emanuela M. Bruscia

**Affiliations:** 1 Departments of Pediatrics, Yale University School of Medicine, New Haven, CT USA; 2 Laboratory Medicine and the Yale Stem Cell Center, Yale University School of Medicine, New Haven, CT USA; 3 Department of Genetics and Genome Sciences, Case Western Reserve University, Cleveland, OH USA; 4 Prolong Pharmaceuticals, South Plainfield, NJ USA; 5 Cellular and Molecular Physiology, Yale University School of Medicine, New Haven, CT USA

**Keywords:** Innate immune cells, Diseases

## Abstract

Overwhelming neutrophilic inflammation is a leading cause of lung damage in many pulmonary diseases, including cystic fibrosis (CF). The heme oxygenase-1 (HO-1)/carbon monoxide (CO) pathway mediates the resolution of inflammation and is defective in CF-affected macrophages (MΦs). Here, we provide evidence that systemic administration of PP-007, a CO releasing/O_2_ transfer agent, induces the expression of HO-1 in a myeloid differentiation factor 88 (MyD88) and phosphatidylinositol 3-kinase (PI3K)/protein kinase B (AKT)-dependent manner. It also rescues the reduced HO-1 levels in CF-affected cells induced in response to lipopolysaccharides (LPS) or *Pseudomonas aeruginosa* (PA). Treatment of CF and muco-obstructive lung disease mouse models with a single clinically relevant dose of PP-007 leads to effective resolution of lung neutrophilia and to decreased levels of proinflammatory cytokines in response to LPS. Using HO-1 conditional knockout mice, we show that the beneficial effect of PP-007 is due to the priming of circulating monocytes trafficking to the lungs in response to infection to express high levels of HO-1. Finally, we show that PP-007 does not compromise the clearance of PA in the setting of chronic airway infection. Overall, we reveal the mechanism of action of PP-007 responsible for the immunomodulatory function observed in clinical trials for a wide range of diseases and demonstrate the potential use of PP-007 in controlling neutrophilic pulmonary inflammation by promoting the expression of HO-1 in monocytes/macrophages.

## Cystic fibrosis: Enzyme boost could limit lung inflammation

The activity of an enzyme that is significantly reduced in cystic fibrosis (CF) could be boosted by an existing drug, reducing lung inflammation and associated tissue damage. Chronic inflammation in CF is currently treated using long-term corticosteroids which may leave patients immuno-suppressed, or high-dose ibuprofen, which is not well tolerated. Scientists hope to find alternative therapies targeting chronic inflammation. Emanuela Bruscia, Caterina Di Pietro (Yale University, New Haven, USA) and co-workers examined the mechanisms of action of the first-in-class drug PP-007 (Prolong Pharmaceuticals®) and assessed its potential for controlling inflammation in CF. Patients with CF have reduced expression of the heme oxygenase-1 enzyme in immune cells called monocytes. In CF mouse models, treatment with PP-007 boosted the expression of this enzyme in circulating monocytes. The treatment reduced levels of proinflammatory proteins and associated lung damage.

## Introduction

Nonresolving pulmonary neutrophilic inflammation is the underlying mechanism of progressive lung disease and premature death in patients with cystic fibrosis (CF)^1^. Despite the role of inflammation in CF lung disease, corticosteroids or high-dose ibuprofen are the only approved long-term treatments for CF airway inflammation. Both treatments are often poorly tolerated^1,2^. Moreover, the use of anti-inflammatory agents in CF is restricted by the risk that they will compromise the patients’ host defense, exacerbating lung infections^3^. CFTR-modulating therapies can dramatically improve the life expectancy of individuals born with CF^4,5^. However, these therapies may not decrease lung hyperinflammation, allowing tissue damage to continue^6^. Thus, there is a need for novel therapeutic approaches that, in combination with CFTR modulators, rescue the abnormal anti-inflammatory regulatory mechanisms and facilitate the resolution of the inflammatory response while maintaining potent antimicrobial host defense.

We have previously shown that CF monocytes and macrophages (MΦs) are hyperresponsive to inflammatory triggers^7,8^ and that the HO-1/CO pathway is inefficiently induced in human and murine CF MΦs in response to inflammatory or infectious triggers^9–11^. HO-1 is a key enzyme that catabolizes heme groups to CO and biliverdin^12–15^. The production of CO as a consequence of HO-1 activity acts as a signaling mediator by binding to divalent metals of heme-containing proteins, regulating their functions^13^. CO promotes the activation of signaling pathways implicated in cell protection, survival, and resolution of the inflammatory response^16^. CO also has a strong positive feedback effect on HO-1 expression^9,17,18^. Indeed, exogenous delivery of controlled doses of CO by inhalation to induce endogenous HO-1 has long been associated with beneficial effects in many inflammatory lung diseases^16,17,19–23^. However, the controlled delivery of nontoxic doses of CO can be challenging. In addition, the delivery of drugs by inhalation in diseases that feature airway mucus obstruction, such as CF, often requires intravenous (IV) therapies to achieve clinical benefit.

Here, we studied the efficacy and mechanism of action of IV administration of PP-007, a polyethylene-glycol-modified (PEGylated) bovine hemoglobin-based CO carrier^24,25^, to control neutrophilic pulmonary inflammation in CF and CF-like mouse models. PP-007 is designed to systemically release CO through oxygen (O_2_) exchange. Pharmacological studies in large animal models and humans have shown that CO is released within 2 h of infusion and exchanged for oxygen, which is then delivered to areas of low oxygen tension^25,26^. This dual mode of action targets inflammation (CO) and hypoxia (O_2_), two complications in muco-obstructive lung diseases. PP-007 is also PEGylated, which enhances the compound’s stability and prolongs its retention in the circulation. PP-007 has been used safely with potential clinical benefit in multiple phase I/II human trials for diseases characterized by increased inflammation, such as sickle cell disease^27,28^, cerebral hemorrhage^29^, and end-stage renal disease^26^, and is currently approved by the FDA for compassionate use^30^. However, the exact mechanism of PP-007 is unknown, and its efficacy in controlling lung hyperinflammation in the context of muco-obstructive lung diseases and respiratory infection has not been previously assessed.

Here, we provide evidence that PP-007 primes monocytes/MΦs to express high levels of HO-1 and that this promotes the resolution of neutrophilic pulmonary inflammation without compromising the clearance of *Pseudomonas aeruginosa* (PA) in mouse models of CF. Ultimately, these studies highlight the relevance of the HO-1/CO pathway in monocytes/MΦs as a therapeutic target in controlling neutrophilic lung inflammation in pathological conditions such as CF.

## Materials and methods

### Chemicals and reagents

**Fig. 1:**
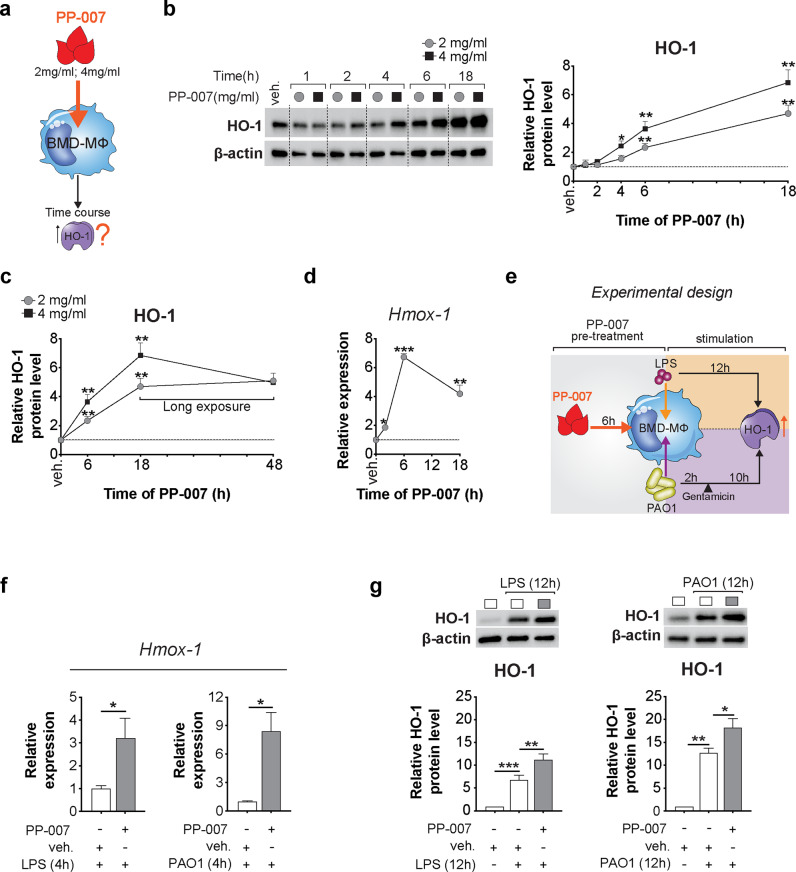
PP-007 is a strong inducer of HO-1 in macrophages. **a** Schematic cartoon showing the treatment regimen. **b**, **c** Representative western blot (WB) and densitometric analysis of HO-1 in WT MΦs treated with 2 mg/ml or 4 mg/ml PP-007 for the time indicated. **d** qPCR of *Hmox-1* in WT MΦs treated with 2 mg/ml PP-007 for the time indicated. **e** Cartoon showing the experimental design: WT BMD-MΦs were pretreated with 2 mg/ml PP-007 for 6 h and then challenged with PA-LPS or live PAO1 (MOI of 10:1) for an additional 12 h. At 2 h after exposure to PAO1, 100 µg/ml gentamicin was added to the medium. **f** qPCR of *Hmox-1* in WT MΦs treated with 2 mg/ml PP-007 for 6 h before the addition of PA-LPS for an additional 4 h. **g** Representative WB and densitometric analysis of HO-1 in WT MΦs pretreated with vehicle or 2 mg/ml PP-007 for 6 h and then challenged with PA-LPS (left) or PAO1 (right). WB and qPCR data are represented as the fold increase over vehicle-treated samples. mRNA levels were normalized to the 18 S level. For WB data, band intensities were normalized to the corresponding β-actin band intensity. Data are represented as the mean ± SEM of at least three biological repeats. Statistical analyses were conducted using a two-tailed unpaired Student’s *t* test with unequal variance: *P ≤ 0.05, **P < 0.01, and ***P < 0.001. In (**b**–**d**), *symbols indicate a statistically significant difference between PP-007-treated and vehicle-treated samples. Cropped blots are displayed, and full-length gels and blots are included in the Supplementary Methods.

*PP-007* was provided by Prolong Pharmaceuticals (South Plainfield, New Jersey) at a concentration of 40 mg/ml, and MINBASE was used as the vehicle in all experiments. PP-007 was directly added to cells at a concentration of 2 mg/ml or 4 mg/ml (50 µl or 100 µl, respectively) as indicated, and the same amount of vehicle was used in all experiments. *Pseudomonas aeruginosa* (PA) LPS (Sigma-Aldrich) was prepared in PBS to generate a 100× stock solution and used at a concentration of 10 µg/mL. The PI3K/AKT inhibitor LY94002 (Cell Signaling Technology) was prepared in DMSO and used at a concentration of 20 μM. The PA strain O1 (PAO1) was grown at 37 °C in lysogeny broth. Overnight cultures of PAO1 were washed and resuspended in antibiotic-free DMEM + 10% FBS, and the bacterial concentration was determined. Bone marrow-derived (BMD)-MΦs were seeded (1 × 10^6^) and allowed to adhere overnight prior to infection with bacteria at a MOI of 10:1. Extracellular bacteria were killed with 100 µg/ml gentamicin (Sigma-Aldrich) after 2 h of exposure to overcome PA replication in the medium and MΦ death, as shown in Fig. 1e.

### Isolation and culture of murine bone marrow-derived and human peripheral blood-derived macrophages

#### Murine macrophages

BM cells were isolated from mice at 6–8 weeks of age. BM collection was performed as described previously^31^. BM cells were flushed from the medullary cavities of the tibias and femurs with DMEM using a 25-G needle under sterile conditions, and the BM suspension was filtered through a 70-µm cell strainer. After overnight culture, the nonadherent cells were differentiated for 7 days in 20 ng/ml recombinant M-CSF (ConnStem Inc., CT, USA). After 7 days, the cells were detached and characterized by flow cytometry (F4/80^+^/MAC-1^+^ population). The day before the experiments, the cells were plated according to the experimental design.

#### Human macrophages

Blood was obtained from healthy donors (HD) or pediatric CF subjects (Supplementary Table 1) during routine clinic visits with informed consent in accordance with protocols approved by the Yale University Medical School Human Investigation Committee (IRB number: 0906005332). Human MΦs were cultured as described previously^32^. Human mononuclear cells were isolated from whole blood by the Ficoll-Paque method (Histopaque 1077 Sigma H8889) and seeded in 24-well plates in RPMI medium supplemented with 10% FBS and 40 ng/ml recombinant human M-CSF (ConnStem Inc., CT, USA). The cells were fed every other day and split 1:1 every 4 days. After 2 weeks, the cells were characterized by flow cytometry (CD14^+^/CD45^+^). Before LPS treatment, cells were washed extensively with PBS.

### Mouse models and in vivo studies

All procedures were performed in compliance with relevant laws and institutional guidelines and were approved by the Yale University Institutional Animal Care and Use Committee. Transgenic CFTR^−/−^ (B6.129P2-KOCftr^tm1UNC^; CF) mice were purchased from The Jackson Laboratory and bred in the Yale University Animal Facility, as previously described^31^. Homozygous AKT1-KO mice were on the C57BL/6 background; they were generated as previously described^33^ and provided by Dr. Patty J. Lee. MyD88-KO and TRIF-KO mice were originally purchased from The Jackson Laboratory. TRIF-KO mice were kindly provided by Dr. Clemente Britto. βENaC-Tg (*B6. Cg-Tg-Scgb1a1-Scnn1b-6608Bouc/J*) mice were developed by the Boucher group^34^ and purchased from The Jackson Laboratory. Hmox-1^fl/fl^ (B6J.129P2-Hmox-1< tm1Mym >) mice were originated at the RIKEN BRC through the National Bio-Resource Project, Japan^35^, and kindly provided by Dr. Wegiel^36^. Hmox-1^fl/fl^ mice were bred with Cx3Cr1-Cre mice (The Jackson Laboratory)^37^, and Cx3Cr1^*Cre/+*^*Hmox-1*^*fl/fl*^ (HO-1^Cx3cr1^) mice were used for experiments.

### In vivo treatment

For in vivo studies, mice were retro-orbitally injected with 1 dose of PP-007 (320 mg/kg) or vehicle. Beginning at 6 h after injection, the mice received three doses of PA-LPS (Sigma L8643, Sigma, St. Louis, MO) over 3 days (one dose daily). PA-LPS (12.5 mg) was administered to the mice with a nebulizer (Pulmo-Aide Compressor; Natallergy, Duluth, GA). A 5-ml aliquot of the solution was nebulized over 15 min. A schema of the LPS administration time points is shown in Fig. 5a.

For a chronic infection model, 1 dose of PP-007 (320 mg/kg) or vehicle was administered intravenously. The PA strain, PA-M57-15, a mucoid strain isolated from the lungs of a patient with CF, was embedded in agarose beads, as described previously^38,39^. Mice were anesthetized with isoflurane via inhalation with a nosecone, and PA-M57-15 was instilled into the right mainstem bronchus at a sublethal dose of 10^5^ viable CFU in 50 μl of PBS using a 20-G x 1” flexible catheter. The total bacterial dose was confirmed after bead preparation by bacterial culture. A schema of the experimental design is shown in Supplementary Fig. 7a.

### Bronchoalveolar lavage fluid (BALF) and lung tissue collection

#### BALF

BALF was collected using standard methods. The BALF was first passed through a 100-μm cell strainer and then centrifuged at 1200 rpm for 10 min. The supernatants were collected and stored in single-use aliquots at −80 °C until analysis, while the cell pellets were resuspended in PBS, counted, and used for flow cytometry for differential cell counting.

For infection experiments, cells were cytospun onto slides (20,000 cells/slide, 2 slides per BAL) and stained with Wright Giemsa. At least 200 cells per slide were classified. Values are expressed as the percentage of each different cell type, and the total numbers of neutrophils, lymphocytes, and macrophages for each sample were calculated.

#### Lung tissues

Lungs were collected via a midline incision from the sternum to the diaphragm, and the blood was removed from the pulmonary circulation by perfusion of PBS supplemented with heparin. After cardiac perfusion, the left lung lobes were inflated with 0.5% low-melting agarose in PBS at constant pressure, harvested, fixed overnight in 10% neutral buffered formalin, and embedded in paraffin. The right lobes were used as follows: The upper lobes were collected in RNAlater and used for RNA extraction, the middle lobes were snap-frozen in liquid nitrogen and used for protein isolation, and the bottom lobes were collected in PBS and used for flow cytometry. Paraffin-embedded tissues were stained with hematoxylin–eosin for morphological analysis or used for immunofluorescence.

### Immunofluorescence staining of lung tissues

Formalin-fixed, paraffin-embedded lung tissue sections were deparaffinized with xylene, rehydrated gradually with graded alcohol solutions, and then washed with deionized water. After antigen retrieval and blocking, the sections were incubated with rabbit polyclonal anti-HO-1 (1:200, Abcam) and rat monoclonal anti-CD68 (1:100, Bio-Rad) antibodies at 4 °C overnight. The sections were washed in PBS and then incubated with a 1:400 dilution of fluorophore-labeled anti-rabbit (Alexa 555) and anti-rat (Alexa 488) antibodies at room temperature for 2 h. DAPI was used for nuclear counterstaining. The slides were then washed with PBS and mounted with mounting medium containing 4′,6-diaminido-2-phenylindole (Vector Laboratories). Images were acquired with a confocal microscope (Leica TCS SP5 Spectral Confocal Microscope). For each experiment, at least six different fields were examined.

### Cytokine quantification

The cytokine concentration in the BALF was assessed by MILLIplex following the manufacturer’s instructions (MCYTOMAG-70K, Millipore). The following cytokines were simultaneously assessed in 50 μl of BALF: IL-1β, IL-6, TNF-α, IL-12p70, macrophage inflammatory protein (MIP)-1α, monocyte chemoattractant protein (MCP)-1, IL-1α, IL-17, IFN-γ-induced protein 10 (IP-10), granulocyte colony-stimulating factor (G-CSF) and chemokine (C-X-C motif) ligand 1 (CXCL1).

### RT–PCR and expression analysis

Cells were lysed in TRIzol, and total RNA was isolated from 1 × 10^6^ cells using an RNA Mini Kit (Qiagen) following the manufacturer’s instructions. Lung tissues were homogenized before RNA isolation. Two hundred microliters of blood was recovered by retro-orbital puncture, and RNA was isolated from white blood cells using a QIAamp Blood Mini Kit (Qiagen). Then, 1 μg of total RNA was reverse transcribed using *SuperScript*™ *II Reverse Transcriptase* (Thermo Fisher) following the manufacturer’s specifications. Real-time PCR analysis was performed with a Bio-Rad iCycler using TaqMan technology with primers and probes purchased from Applied Biosystems (Life Technology). The copy number was normalized to 18S rRNA levels, and relative expression with comparison to untreated cells was calculated by the ∆∆Ct method.

### Protein isolation and western blotting

Cold RIPA lysis buffer (Cell Signaling) containing 1 mM phenylmethanesulfonyl fluoride (PMSF) and protease and phosphatase inhibitor cocktails (Roche Diagnostics) was added to cells or lung tissue lysates. After 30 min of incubation on ice, the lysates were centrifuged, and the supernatants were recovered. Equal amounts of protein were separated by electrophoresis on 4–15% Mini PROTEAN Gels (Bio-Rad Laboratories, CA), transferred to nitrocellulose membranes (Bio-Rad Laboratories, CA), and incubated with primary antibodies overnight at 4 °C. HRP-conjugated IgG secondary Abs (1:2000; Santa Cruz Biotechnology) and an Amersham ECL Plus Western blotting system (GE Healthcare Bio-Sciences) were used for detection. The following primary antibodies were used: rabbit polyclonal anti-HO-1 (1:1000, Abcam), rabbit polyclonal anti-AKT and anti-pAKT (1:1000, Cell Signaling Technology), and rabbit-HRP anti-actin (1:5000, Santa Cruz). The chemiluminescence imaging system ChemiDoc (Bio-Rad) and Image Lab software (Bio-Rad) were used for image acquisition and signal quantification. Relative protein expression was normalized to β-actin expression. The pAKT/AKT ratio was calculated as follows: first, each WB band intensity was normalized to the corresponding β-actin intensity. From the normalized values, we calculated the fold increase relative to control samples. From these values, the pAKT/AKT ratio was calculated. Images have been cropped for presentation. Full-size images are presented in the Supplementary Methods.

### Flow cytometry and cell sorting

As previously described, the inferior lung lobes collected in PBS were used for flow cytometry. The lobes were transferred into C-tubes (Miltenyi, Auburn, CA) and processed in digestion buffer with a GentleMACS dissociator (Miltenyi) according to the manufacturer’s instructions. The homogenized lung tissues were passed through a 100-μm cell strainer to obtain a single-cell suspension. The remaining red blood cells were lysed using BD Pharm Lyse (BD Biosciences, San Jose, CA). After the cells were counted, 1 × 10^6^ cells were used for staining, while up to 1 × 10^7^ lung cells were used for sorting. The cells were stained with the viability dye Aqua (Invitrogen), incubated with FcBlock (BD Biosciences), and then stained with a standard panel of immunophenotyping antibodies for 30 min at 4 °C. After staining, the cells were washed and fixed with 2% paraformaldehyde in PBS. Data were acquired on a BD LSR II flow cytometer using BD FACSDiva software, and data analyses were performed using FlowJo software (TreeStar, Ashland, OR). Cell sorting was performed on a FACSAria II instrument (BD Biosciences) with the same configuration as the LSR II. Cell populations were identified using a sequential gating strategy, and the percentage of cells in the live/singlets gate was multiplied by the number of live cells (after trypan blue exclusion) to obtain an absolute live-cell count. The following antibodies were used for flow cytometry and cell sorting: anti-CD45-BUV395 (BD Horizon, clone 30-F11), anti-CD11b-PE-Cy7 (Invitrogen, clone M1/70), anti-CD64-APC (BioLegend, clone X54-5/7.1), anti-CD24-V-450 (Invitrogen, clone M1/69), anti-Ly6C-BV605 (BD Horizon, clone AL-31), anti-CD11c-BV-711 (BD Horizon, clone HL3), anti-Ly6-G-AF-700 (BD Pharmingen, clone 1A8), anti-Siglec-F-PerCP-Cy5.5 (BD Pharmingen, clone E50-2440), anti-MHC-II-APC/Fire (BioLegend, clone M5/114.15.2), anti-CD4-PerCP-Cy5.5 (BD Pharmingen, clone RM4-5), anti-CD8a-FITC (BioLegend, clone 53-6.7), and anti-B220-PE-Cy7 (BD Pharmingen, clone RM4-5).

### Statistical analysis

All data were analyzed using GraphPad Prism 8.0 software (GraphPad, San Diego, CA). The statistical significance of data was assessed by a two-tailed unpaired Student’s *t* test with Welch’s correction. For in vivo experiments, data that were not normally distributed were further confirmed with a nonparametric Wilcoxon rank-sum test. All values are presented as the mean ± SEM. A *P* value ≤0.05 was considered statistically significant: *P ≤ 0.05, **P < 0.01, and ***P < 0.001.

## Results

### PP-007 is a strong inducer of HO-1 in macrophages

We first tested whether treatment with PP-007 induces HO-1 expression in MΦs in vitro. Murine wild-type (WT) BMD-MΦs were treated with two clinically relevant PP-007 concentrations (2 and 4 mg/ml) under steady-state conditions (e.g., absence of inflammatory/infectious triggers), and HO-1 levels were assessed over time (Fig. 1a). PP-007 increased HO-1 protein levels, with the highest level observed 18 h after treatment (Fig. 1b). The effect of PP-007 on HO-1 expression was more pronounced with the 4 mg/ml dose (Fig. 1b). When we repeated the study with a longer time course, we found that HO-1 protein levels were more stable between 18 and 48 h in cells treated with the 2 mg/ml dose (Fig. 1c). Therefore, for subsequent experiments, we chose the 2 mg/ml dose. At this dose, the peak of *Hmox-1* (which encodes the HO-1 protein) mRNA expression in MΦs was observed at 6 h (sevenfold increase compared to vehicle-treated cells), and this expression remained elevated (fourfold) for up to 18 h (Fig. 1d). The effect of PP-007 on HO-1 induction was also evaluated in the context of infection. Namely, WT BMD-MΦs pretreated with 2 mg/ml PP-007 for 6 h (time of the highest *Hmox-1* expression) had increased HO-1 expression and doubled HO-1 protein levels in response to PA-lipopolysaccharide (LPS) or live PA (PAO1) compared with WT BMD-MΦs given vehicle pretreatment (Fig. 1e–g). Taken together, these data show that PP-007 is a potent inducer of the HO-1/CO pathway in MΦs.

### PP-007 requires the combined activation of MyD88 and PI3K/AKT for optimal induction of HO-1

**Fig. 2:**
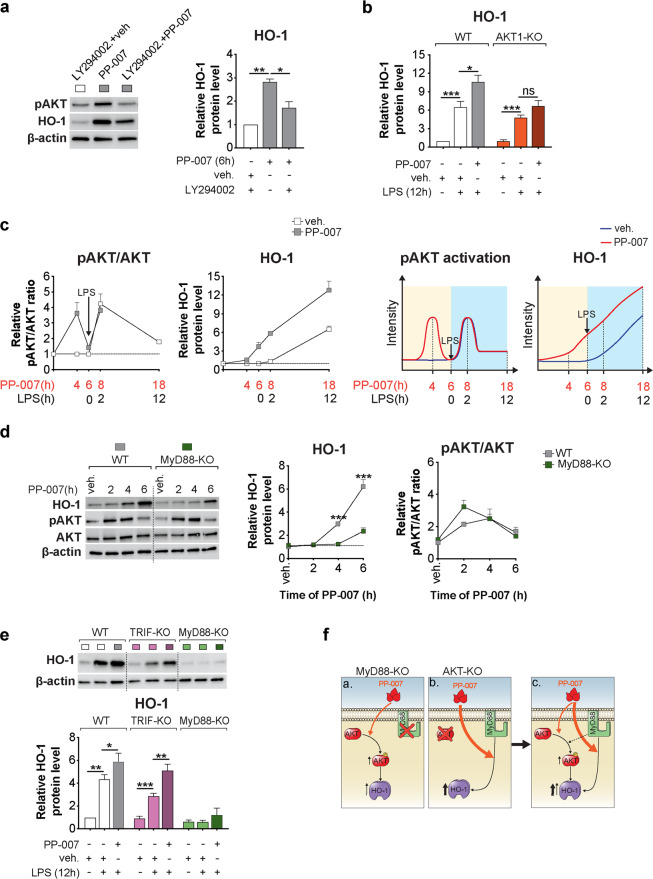
PP-007 requires the combined activation of MyD88 and PI3K/AKT for optimal induction of HO-1. **a** Representative WB of phospho-AKT (pAKT, serine 473) and HO-1 and densitometric analysis of HO-1 in WT MΦs pretreated for 12 h with 20 μM LY294002 or DMSO and then exposed to vehicle or 2 mg/ml PP-007 for an additional 6 h, as indicated. **b** Densitometric analysis of HO-1 in WT and AKT1-KO MΦs pretreated with vehicle or 2 mg/ml PP-007 for 6 h and then challenged with PA-LPS for 12 h. **c** Densitometric analysis of pAKT, total AKT, and HO-1 in murine WT MΦs treated with vehicle or PP-007 at different time points before and after the addition of PA-LPS (left). Schematic representation of the effects of PP-007 on AKT phosphorylation and HO-1 induction in MΦs before and after LPS addition (right). **d** Representative WB and densitometric analysis of HO-1, pAKT, and total AKT in WT and MyD88-KO MΦs treated with 2 mg/ml PP-007 for the time indicated. Time 0 indicates samples treated with vehicle. **e** Representative immunoblot and densitometric analysis of HO-1 in WT, TRIF-KO, and MyD88-KO MΦs pretreated with vehicle or 2 mg/ml PP-007 for 6 h and then challenged with PA-LPS for 12 h. **f** Schematic representation of the mechanism of action of PP-007: activation of the adaptor MyD88, but not PI3K/AKT signaling activation alone, is strictly required for robust PP-007-mediated induction of HO-1 (a, b). The combination of MyD88 and PI3K/AKT signaling activation led to the maximum induction of HO-1 (c). For WB data, band intensities were normalized to the corresponding β-actin band intensity, and the rate of AKT phosphorylation is shown as the ratio of phosphoprotein to total protein. Unless otherwise indicated, data are presented as the fold increase relative to WT vehicle-treated samples. Data are represented as the mean ± SEM of at least three biological repeats. Statistical analyses were conducted using a two-tailed unpaired Student’s *t* test with unequal variance: *P ≤ 0.05, **P < 0.01, and ***P < 0.001. In (**d**), * symbols indicate a statistically significant difference between different genotypes. Cropped blots are displayed, and full-length gels and blots are included in the Supplementary Methods.

We have previously shown that the PI3K/AKT pathway is an upstream regulator of HO-1 induction in MΦs challenged with LPS or PA^10^. Thus, we investigated whether the mechanism of action of PP-007 relies on the activation of this pathway. In the absence of infectious triggers, PP-007 transiently enhanced AKT phosphorylation in MΦs, with the highest AKT phosphorylation level observed at 4 h after PP-007 exposure and with the return to baseline observed within 6 h (Supplementary Fig. 1a). WT MΦs preincubated with a specific PI3K inhibitor (LY29002) showed 50% less induction of HO-1 in response to PP-007 (Fig. 2a). The requirement for PI3K/AKT for optimal HO-1 induction was further demonstrated by the inability of PP-007 to enhance HO-1 above LPS-induced levels in BMD-MΦs from AKT1-KO mice (Fig. 2b). This suggests that PI3K/AKT activation is partially required for the PP-007-dependent induction of HO-1. We performed a time-course study to clarify the correlation between PP-007-dependent PI3K/AKT activation and HO-1 induction during LPS exposure. PP-007 pretreatment induced a temporary increase in AKT phosphorylation, which led to HO-1 induction. Subsequent treatment with LPS reactivated the PI3K/AKT pathway, leading to an additive increase in the final HO-1 cellular levels (Fig. 2c).

We then investigated whether the TLR4 complex is required for the mechanism of action of PP-007 in BMD-MΦs. Under steady-state conditions, the TLR4 adaptor protein MyD88 (Fig. 2d) (but not TRIF (Supplementary Fig. 1b)) was required for robust induction of HO-1 in response to PP-007 treatment but not for PI3K/AKT activation (Fig. 2d and Supplementary Fig. 1b). In response to LPS, MyD88 (but not TRIF) was also required for the induction of HO-1 activation and effectiveness of PP-007 (Fig. 2e). Consistent with published data^40^, MyD88 (but not TRIF) was partially required for the activation of the PI3K/AKT pathway in response to LPS (Supplementary Fig. 1c). Because Nrf2 is a major transcriptional regulator of *Hmox-1* expression, we tested whether PP-007 treatment of MΦs induces other Nrf2 target genes (e.g., NADPH quinone oxidoreductase 1, *Nqo1,* and the glutamate-cysteine ligase catalytic subunits *Gclc* and *Gclm*). While PP-007 rapidly (2 to 6 h) induced *Hmox-1* expression, *Nqo1*, *Gclc*, and *Gclm* were also induced but at lower levels and at a later time point (18 h) (Supplementary Fig. 1d, left). On their own, neither LPS nor PP-007+LPS led to robust induction of Nrf2 target genes, in contrast to the induction observed for *Hmox-1* (Supplementary Fig. 1d, right), suggesting that PP-007 may work by activating transcription factors other than Nrf2.

Taken together, these data establish that MyD88 is strictly required for the mechanism of action of PP-007 and that combined PI3K/AKT activation leads to the maximum induction of HO-1 (Fig. 2f).

### PP-007 rescues the PI3K/HO-1 axis in CF macrophages and decreases the hyperinflammatory response to LPS

**Fig. 3:**
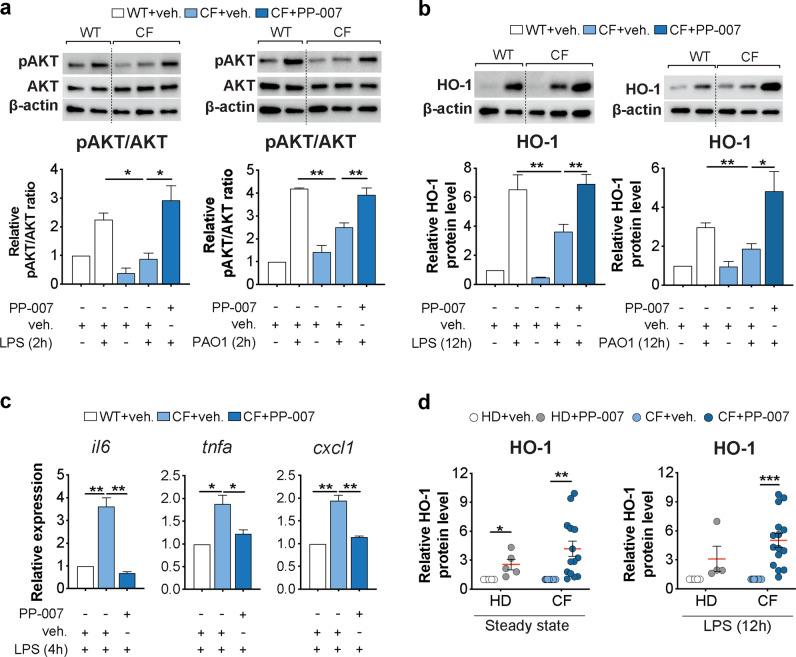
PP-007 rescues the PI3K/HO-1 axis in CF macrophages and decreases the hyperinflammatory response to LPS. **a**, **b** Representative WB and densitometric analysis of pAKT, total AKT, and HO-1 in CF MΦs pretreated with vehicle or 2 mg/ml PP-007 for 6 h before the addition of PA-LPS (left) or PAO1 (right) for an additional 2 h (**a**) or 12 h (**b**), as shown in Fig. 1e. WT MΦs were pretreated with the vehicle and used as a control. **c** qPCR of *il6*, *tnfa*, and *cxcl1* in CF MΦs pretreated with vehicle or 2 mg/ml PP-007 for 6 h before the addition of PA-LPS for an additional 4 h. WT MΦs pretreated with the vehicle and exposed to PA-LPS were used as a control. **d** Densitometric analysis of HO-1 in PBD MΦs from HD (*n* = 5) and CF patients (*n* = 14) treated for 18 h with vehicle or 2 mg/ml PP-007 (left). Densitometric analysis of HO-1 in PBD MΦs from HD (*n* = 4) and CF patients (*n* = 15) pretreated for 6 h with 2 mg/ml PP-007 and then exposed to PA-LPS for 12 h (right). WB and qPCR data are shown as the fold increase relative to WT vehicle-treated samples. mRNA levels were normalized to the 18S level. For WB data, band intensities were normalized to the corresponding β-actin band intensity, and the rate of AKT phosphorylation is shown as the ratio of phosphoprotein to total protein. Data are represented as the mean ± SEM and, unless otherwise indicated, are the result of three biological repeats. Statistical analyses were conducted using a two-tailed unpaired Student’s *t* test with unequal variance: *P ≤ 0.05, **P < 0.01, and ***P < 0.001. Cropped blots are displayed, and full-length gels and blots are included in the Supplementary Methods. The genotypes of CF patients enrolled in this study are listed in Supplementary Table 1.

We have previously demonstrated that defective induction of the PI3K-HO-1 axis in activated CF MΦs leads to hyperinflammatory responses downstream of TLR4 signaling^10,15^. Thus, we tested the effects of PP-007 pretreatment of CF MΦs responding to bacterial infection to determine whether prior correction of the HO-1 defect restores a wild-type inflammatory response. CF BMD-MΦs were pretreated with 2 mg/ml PP-007 for 6 h and then challenged with live PA (PAO1) or PA-LPS (as shown in Fig. 1e). PP-007 pretreatment rescued PI3K signaling to levels comparable to those in WT MΦs (Fig. 3a) and allowed normal induction of HO-1 (Fig. 3b and Supplementary Fig. 2a, b). Moreover, pretreatment with PP-007 decreased the expression of proinflammatory cytokines *(Il6*, *Tnfα*, and *Cxcl1)* in CF MΦs challenged with LPS, as was observed in WT MΦs (Fig. 3c). Consistent with these data for murine cells, we found that PP-007 potently induced HO-1 in monocyte-derived MΦs obtained from either healthy donors (HD) or patients with CF (Supplementary Table 1) under steady-state conditions and in response to LPS (Fig. 3d).

Thus, PP-007 pretreatment rescues the blunted PI3K/AKT activation observed in CF MΦs in response to infectious triggers and allows the appropriate induction of HO-1, normalizing the inflammatory response by these cells.

### Systemic delivery of PP-007 induces HO-1 expression in lung monocytes/macrophages

**Fig. 4:**
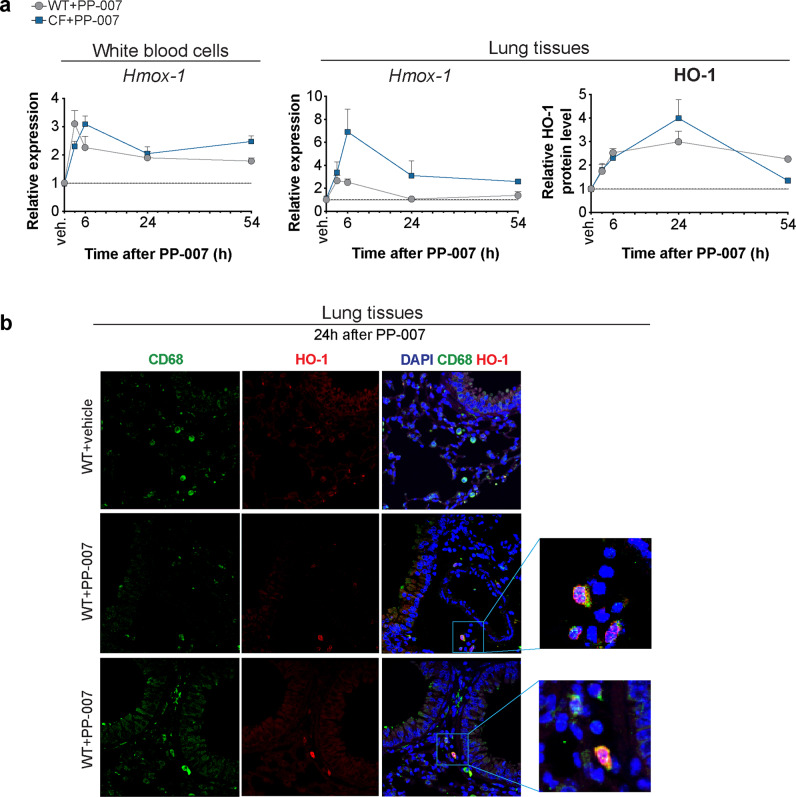
Systemic delivery of PP-007 induces HO-1 expression in lung macrophages. **a** qPCR of *Hmox-1* in white blood cells and lung tissues and densitometric analysis of HO-1 protein levels in lung tissues from WT and CF mice at different time points after PP-007 treatment. Time 0 indicates values for vehicle-treated WT and CF mice. Data are shown as the fold increase in PP-007-treated mice relative to vehicle-treated mice. The graphs show the mean ± SEM and are a combination of two independent experiments with 2–5 mice/time point for each genotype. **b** Representative immunofluorescence (IF) staining for CD68 (green), HO-1 (red), and DAPI (blue) in lung tissues from WT mice 24 h after PP-007 treatment. Merged images and the magnifications of areas of interest are shown on the right. Yellow staining indicates CD68 and HO-1 colocalization.

IV administration of PP-007 once or twice at a clinically relevant dose (320 mg/kg)^26–28^ efficiently induced HO-1 in the lung tissues of WT mice 6 h after treatment. This was maintained for up to 24 h. Treatment with a second dose of PP-007 (24 h after the first) did not increase HO-1 levels above those observed in response to a single dose (Supplementary Fig. 3a, b). Thus, subsequent experiments were performed using a single PP-007 administration. Upon PP-007 infusion, HO-1 was induced in circulating white blood cells and lung tissues as early as 3 h and as late as 54 h in both WT mice and CF mice (Fig. 4a). The highest HO-1 expression level was observed between 3 h and 6 h after PP-007 treatment, while the HO-1 protein levels in lung tissues peaked at 24 h post-treatment (Fig. 4a). Lung tissues from mice treated with PP-007 but not those from mice treated with vehicle were populated by MΦs with high HO-1 expression (CD68^+^) localized in the lung parenchyma (Fig. 4b). To test whether these MΦs with high HO-1 expression originated from the circulation (monocyte-derived macrophages, mo-Ms) or were tissue-resident cells, we assessed HO-1 expression in sorted lung monocyte/MΦ populations from CF mice treated with vehicle or PP-007. We found that PP-007 administration induced *Hmox-1* expression in Ly6C^+^ mo-Ms and interstitial macrophages (IMs) but not in Ly6C^−^ mo-Ms or alveolar MΦs (AMs), without affecting their respective percentages, compared to vehicle treatment (Supplementary Fig. 3c–e). Together, these data suggest that the systemic delivery of PP-007 induces HO-1 expression in subpopulations of lung monocytes/MΦs.

### Systemic delivery of PP-007 reduces the inflammatory response in CF lungs

**Fig. 5:**
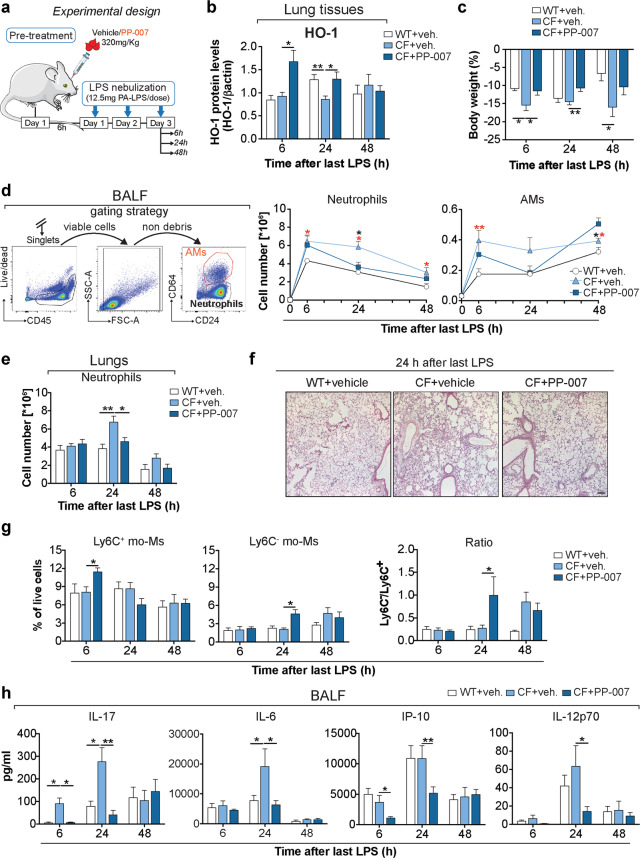
Systemic delivery of PP-007 reduces the inflammatory response in CF lungs. **a** Schematic cartoon showing the treatment regimen: CF mice were retro-orbitally injected with 1 dose of PP-007 (320 mg/kg) or vehicle, while WT mice were injected with vehicle and used as a control. At 6 h after injection, mice received three doses of 12.5 mg of PA-LPS over 3 days (one dose per day) and were analyzed at 6, 24, and 48 h after the last LPS nebulization. **b** Densitometric analysis of HO-1 in lung lysates. The intensities of HO-1 immunoreactive signals were measured and normalized to the β-actin intensity. **c** Weight loss as a percentage of body weight. **d** Differential cell counts in the bronchoalveolar lavage fluid (BALF) of WT and CF mice treated with vehicle and CF mice treated with PP-007 at the time indicated. The left panel shows the gating strategy used to distinguish alveolar macrophage and neutrophil populations in the BALF based on the expression of CD64. **e** Neutrophil numbers in lung tissues. **f** Representative hematoxylin–eosin staining of paraffin-embedded lung tissues 24 h after the last LPS treatment. Magnification: ×10; scale bar: 100 µm. **g** Percentages of Ly6C^+^ and Ly6C^−^ mo-Ms in the live/CD45^+^ gate based on the gating strategy shown in Supplementary Fig. 3. **h** Cytokine concentration in the BALF. For (**e**), neutrophils in the lungs were identified using the sequential gating strategy shown in Supplementary Fig. 3. The percentage of cells in the live/singlets gate was then multiplied by the number of live cells to obtain an absolute live-cell count. AMs alveolar macrophages, mo-Ms monocyte-derived macrophages. The graphs show the mean ± SEM. A detailed list of the mice used in each experiment is included in Supplementary Table 2. In (**d**), the red symbol (*) indicates a statistically significant difference between the vehicle-treated WT and CF groups. The black symbol (*) indicates a statistically significant difference between the vehicle-treated CF and PP-007-treated CF groups. Statistical analyses were conducted using a two-tailed unpaired Student’s *t* test with unequal variance: *P ≤ 0.05, **P < 0.01, and ***P < 0.001.

Next, we tested the effects of PP-007 in the context of pulmonary inflammation. Since the maximum induction of HO-1 was observed between 3 and 6 h after systemic PP-007 administration (Fig. 4a), WT and CF mice were pretreated intravenously (IV) with a single dose of PP-007 (320 mg/kg) or vehicle alone for 6 h and then nebulized with PA*-*LPS for three consecutive days. Mice were sacrificed at 6 h, 24 h, and 48 h after the last LPS nebulization, and bronchoalveolar lavage fluid (BALF) samples and lung tissues were collected (Fig. 5a and Supplementary Table 2). At 6 h after the last dose of LPS, HO-1 levels were increased in the lung tissues of PP-007-treated CF mice compared with those of vehicle-treated control mice. These levels started declining at 48 h (Fig. 5b). Consistent with the previous studies^10,31,39^, CF mice challenged with LPS showed significantly more weight loss than WT mice and failed to regain weight 48 h after the last LPS nebulization. In contrast, PP-007 treatment rescued the severity of weight loss in CF mice to a degree comparable to that in WT mice (Fig. 5c). In all three groups of experimental mice, the total BALF cell number at 6 h after the last dose of LPS was higher than that in the untreated group (0 h), and the BALF cells were predominantly neutrophils, as determined by flow cytometry (Fig. 5d). In line with the previous findings^31^, vehicle-treated CF mice showed a significantly higher number of neutrophils (and MΦs at 6 h) in the BALF than vehicle-treated WT mice at all time points (Fig. 5d and Supplementary Fig. 4a). The number of neutrophils in the lung parenchyma was also elevated in vehicle-treated CF mice compared to WT mice at 24 h post-LPS treatment (Fig. 5e, f). Pretreatment with PP-007 did not affect the number of neutrophils in CF mice during the initial response to LPS. However, it led to significant decreases (twofold reduction) in the numbers of neutrophils in the BALF and lung tissues at 24 h after LPS challenge, comparable to the numbers observed in vehicle-treated WT mice (Fig. 5d–f). PP-007 treatment led to a significantly higher number of alveolar MΦs in CF mice at 48 h than vehicle treatment, suggesting faster recovery to homeostatic lung conditions (Fig. 5d). In the lung tissues of PP-007-treated CF mice, the percentage of Ly6C^+^ mo-Ms (but not Ly6C^-^ mo-Ms) was elevated at the early time point. However, the percentage of Ly6C^-^ mo-Ms increased over time at the expense of Ly6C^+^ cells, suggesting a phenotypic switch toward an anti-inflammatory profile (Fig. 5g). In addition, the numbers of IMs and Ly6C^+^ mo-Ms, which were higher in the lung tissues of CF mice than in those of WT mice at 24 h post-LPS, were restored by pretreatment with PP-007 to the levels observed in WT mice (Supplementary Fig. 4b). The numbers of T (CD4^+^ and CD8^+^) and B lymphocytes in the lung tissues of CF mice were comparable to those of WT controls. However, activated CD4^+^ T lymphocytes (CD69^+^) were elevated in the lungs of vehicle-treated CF mice compared to those of WT mice and were normalized by treatment with PP-007 (Supplementary Fig. 4c). In line with the data suggesting that PP-007 improves the resolution of the CF lung hyperinflammatory response caused by LPS treatment, the BALF concentrations of the immunomodulatory cytokines IL-6, IL-17, IL-12p70 and IP-10 were lower in PP-007-treated CF mice than in vehicle-treated CF control mice and comparable to those in vehicle-treated WT mice at 24 h post-LPS exposure (Fig. 5h and Supplementary Fig. 4d).

We also tested the drug in a βENaC-Tg mouse model that recapitulates the mucus airway obstruction observed in CF and obstructive lung disease^34^. Similar to observations in CF-KO mice, PP-007 treatment was efficient in reducing the levels of lung neutrophils and BALF proinflammatory cytokines (i.e., IL-6, IL-17, IL-12p70, and IP-10) in βENaC-Tg mice exposed to LPS (Supplementary Fig. 5a–c).

PP-007 treatment did not modulate the magnitude of the inflammatory response in WT mice, suggesting that PP-007 helps restore a controlled and balanced inflammatory response to LPS in conditions of dysregulated immunity, as in CF lung tissues. Moreover, PP-007 did not have an immunosuppressive effect in the already balanced tissue environment of WT lungs (Supplementary Fig. 6a–e).

The greatest concern related to using anti-inflammatory agents in CF is that they may compromise host defense and exacerbate PA infections^41^. Therefore, we assessed whether PP-007 leads to worsening of PA infection using an agarose-bead model of chronic PA endobronchial infection^38,39^. CF mice were treated with a single dose of PP-007 (320 mg/kg) or vehicle 6 h before infection with PA embedded in agarose beads. Then, the mice were sacrificed at 3 days post-infection (72 h) (Supplementary Fig. 7a). Mice pretreated with PP-007 were not detrimentally affected in this experimental setting, as indicated by monitoring major changes in weight loss and BALF cell number (Supplementary Fig. 7b, c). Importantly, PA colony-forming units (CFUs) measured in both lung and BALF samples were comparable between vehicle-treated WT and CF mice and PP-007-treated mice, demonstrating that infection was not worsened by PP-007 treatment (Supplementary Fig. 7d).

Taken together, these data show that PP-007 pretreatment induces HO-1 in lung monocyte-derived MΦs, which helps to restore a balanced inflammatory response to LPS in CF lungs without compromising host defense against PA.

### The mechanism of action of PP-007 relies on the induction of HO-1 in monocytes/MΦs to resolve lung inflammation in vivo

**Fig. 6:**
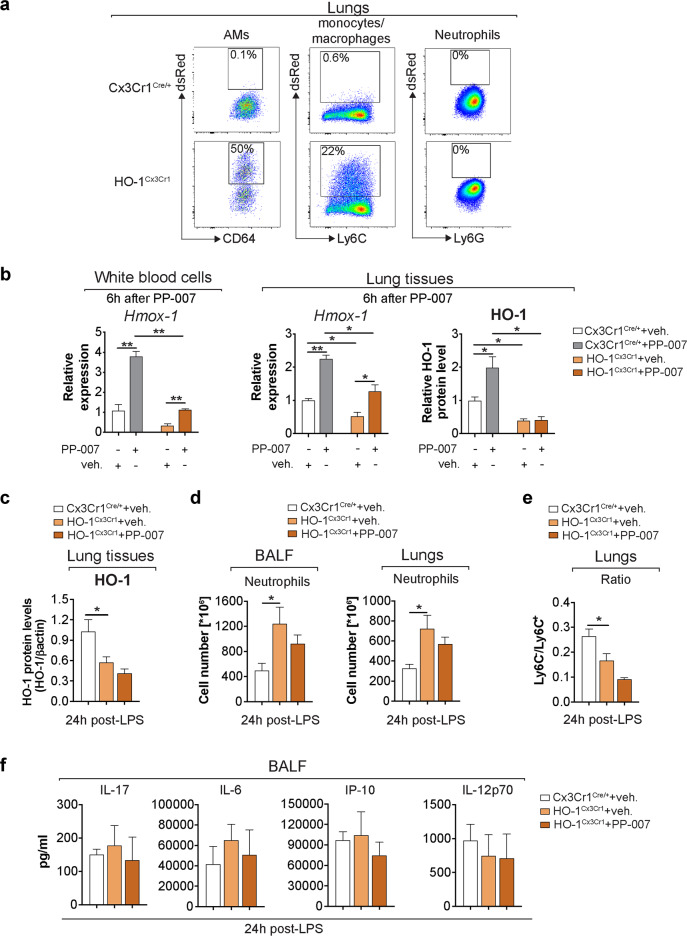
The mechanism of action of PP-007 relies on the induction of HO-1 in monocytes/macrophages to resolve lung inflammation in vivo. **a** Representative dot plots displaying the percentages of DsRed+ alveolar macrophages (AMs; CD11c^+^CD64^+^), neutrophils (CD11c^−^CD11b^+^CD24^+^Ly6G^+^), and other monocytes/macrophages (gran^-^CD11c^-^CD11b^+^) in the lung tissues of Cx3Cr1^Cre/+^ (top panel) and HO-1^Cx3Cr1^ (bottom panel) mice 24 h after LPS treatment. **b** qPCR of *Hmox-1* in white blood cells and lung tissues and densitometric analysis of HO-1 protein levels in lung tissues from Cx3Cr1^Cre/+^ and HO-1^Cx3Cr1^ mice at 6 h after PP-007 treatment (320 mg/kg). Data are shown as the fold increase relative to vehicle-treated Cx3Cr1^Cre/+^ mice. HO-1^Cx3Cr1^ mice were pretreated with vehicle (*n* = 3) or PP-007 (*n* = 4) and then nebulized with PA-LPS as shown in Fig. 5a. Cx3Cr1^Cre/+^ mice (*n* = 3) were pretreated with vehicle and used as a control. **c** Densitometric analysis of HO-1 in lung tissue lysates. The intensities of HO-1 immunoreactive signals were measured and normalized to the β-actin intensity. **d** Numbers of neutrophils in the BALF and lung parenchyma assessed by flow cytometry. **e** Ratio of the percentage of Ly6C^-^ to Ly6C^+^ mo-Ms in the lungs based on the gating strategy shown in Supplementary Fig. 3. **f** Cytokine concentration in the BALF. The graphs show the mean ± SEM. Statistical analyses were conducted using a two-tailed unpaired Student’s *t* test with unequal variance: *P ≤ 0.05.

To demonstrate that the mechanism of action of PP-007 relies on the priming of monocytes/MΦs to express high levels of HO-1 in vivo, we used mice in which HO-1 is specifically knocked out in monocytes/MΦs (HO-1^Cx3Cr1^)^36,42^. Upon induction of HO-1 expression, these mice instead express DsRed fluorescent protein and, therefore, allow tracking of the efficiency and specificity of HO-1 deletion in monocyte/MΦ lung populations using flow cytometry. Lung monocytes/MΦs but not neutrophils exhibited red fluorescence, validating the model (Fig. 6a). In the steady-state, HO-1^Cx3Cr1^ mice had lower HO-1 RNA expression in white blood cells and lung tissues as well as lower protein levels in lung tissues than Cx3Cr1^Cre/+^ controls. PP-007 treatment induced some HO-1 RNA expression, but the overall levels remained significantly lower than those in control mice. The residual amount of HO-1 observed in HO-1^Cx3Cr1^ mice might be ascribed to other cell types. Notably, treatment with PP-007 completely failed to increase HO-1 protein levels in the lung tissues of HO-1^Cx3Cr1^ mice (Fig.  6b). These data confirm that the majority of HO-1 in the lung tissues of PP-007-treated mice originates from monocytes/MΦs. To test whether the PP-007-mediated induction of HO-1 in monocytes/MΦs is responsible for the reduction in lung inflammation induced by LPS, which we reported in CF mice (Fig. 5), HO-1^Cx3Cr1^ mice were pretreated with PP-007 or vehicle alone for 6 h and then nebulized with PA*-*LPS. The mice were sacrificed at 24 h after the last LPS nebulization (as in Fig. 5a). Vehicle-treated HO-1^Cx3Cr1^ mice showed 50% less induction of HO-1 in lung tissues than vehicle-treated Cx3Cr1^Cre/+^ controls. Importantly, the HO-1 levels in lung tissues from HO-1^Cx3Cr1^ mice treated with PP-007 did not increase (Fig. 6c). Consistent with the anti–inflammatory role of HO-1-expressing monocytes/MΦs, vehicle-treated HO-1^Cx3Cr1^ mice showed significantly higher numbers of neutrophils in the BALF (threefold) and lung parenchyma (twofold) than vehicle-treated Cx3Cr1^Cre/+^ mice at 24 h post-LPS treatment (Fig. 6d). Notably, PP-007 treatment failed to reduce the numbers of neutrophils in the BALF and lung tissues of HO-1^Cx3Cr1^ mice. HO-1^Cx3Cr1^ mice, compared with Cx3Cr1^Cre/+^ controls, also showed an elevated number of Ly6C^+^ mo-Ms but not Ly6C^−^ mo-Ms in the lungs at 24 h post-LPS treatment. This ratio was not altered by PP-007 treatment (Fig. 6e). Finally, the BALF concentrations of the immunomodulatory cytokines IL-6, IL-17, IL-12p70, and IP-10 were similar between vehicle-treated Cx3Cr1^Cre/+^ and HO-1^Cx3Cr1^ mice and were not affected by PP-007 treatment (Fig. 6f). This suggests that in the context of CF lung disease, the CF lung tissue environment drives the dysregulated production of immunomodulatory cytokines and that, as observed in WT lungs (Supplementary Fig. 6a–d), PP-007 helps restore the controlled and balanced production of immunomodulatory cytokines in response to LPS in conditions of global dysregulated immunity, as seen in CF lung tissues.

In summary, these data show that monocyte/MΦ populations are the major source of HO-1, which is required for controlling lung neutrophil infiltration in response to infection. Moreover, this model demonstrates that the mechanism of action of PP-007 in vivo relies on the induction of HO-1 in monocytes/MΦs.

## Discussion

Degradation of heme groups by HO enzymes is the only mammalian pathway known to produce CO. This gaseous molecule is toxic at relatively high concentrations. However, at physiological concentrations, CO has strong cytoprotective, anti-inflammatory, antioxidant, and bactericidal properties and provides positive feedback for HO-1 induction^17,19^. As such, the HO-1/CO pathway has been extensively investigated in the context of diseases, and it is considered a major target for drug development^17,19^.

We investigated the efficacy of PP-007 as a delivery system for CO and its potential to reduce tissue hyperinflammation by inducing HO-1 expression. Multiple pharmacological, toxicological, and clinical studies have demonstrated that PP-007 is safe for delivering controlled doses of CO in humans^24,25^. In addition, encouraging data from phase I/II clinical trials support the efficacy of this drug in controlling inflammation in several clinical conditions, including sickle cell disease, reperfusion injury, and cerebral hemorrhage^24–29^. However, hyperinflammation is also a major driver of irreversible lung tissue damage in many chronic lung inflammatory diseases. Thus, in this study, we tested this drug in the context of a CF model of lung hyperinflammation and deepened our understanding of the mechanism of action of PP-007.

The studies presented herein demonstrate that PP-007 is a strong inducer of HO-1 in human and murine MΦs (Figs. 1 and 3). By increasing HO-1 levels, the mechanism of action of PP-007 involves activation of the MyD88 and PI3K/AKT signaling pathways (Fig. 2), which we previously showed to be dysregulated in CF MΦs^10^ and found to be normalized by the drug (Figs. 2 and 3). The systemic delivery of a single clinically relevant dose of PP-007 in mouse models of CF lung hyperinflammation was shown to induce HO-1 expression in circulating blood monocytes, which emigrate and repopulate lung MΦ populations in response to LPS (Figs. 4 and 5g). This is consistent with the fact that monocytes/MΦs play a critical role in the resolution of inflammation, they rely on abundant induction of HO-1 to control inflammation, and dysregulation of MΦs results in hyperinflammation in CF, asthma, COPD, and fibrotic lung diseases^8,43,44^.

Interestingly, the monocytes expressing high levels of HO-1 were shown to be LyC6^+^ (Supplementary Fig. 3d), suggesting that these initially proinflammatory cells are primed to acquire an anti-inflammatory phenotype once they migrate into the tissue. In support of this, in PP-007-treated CF mice, the percentage of Ly6C^−^ mo-Ms increased over time at the expense of Ly6C^+^ mo-Ms in response to LPS nebulization (Fig. 5g), suggesting the acquisition of an anti-inflammatory phenotype^45^. HO-1 was also highly induced in IMs, a population of MΦs that differentiate from circulating monocytes in response to lung infections and with known immunomodulatory functions^46^. These data extend previous studies showing that HO-1 expression modulates MΦ polarization from a proinflammatory phenotype to an anti–inflammatory phenotype, protecting against liver ischemia–reperfusion injury in mouse models^42^.

Our studies with HO-1^Cx3Cr1^ mice (Fig. 6) confirm our central hypothesis that the mechanism of action of PP-007 relies on the induction of HO-1 in monocytes/ MΦs, decreasing the number of inflammatory Ly6C^+^ monocytes in lung tissues induced by LPS. Importantly, this switch in the monocyte population phenotype is associated with a decreased number of lung neutrophils, consistent with the contribution of inflammatory monocytes to facilitating neutrophil migration to the lungs during inflammation^47^. Thus, our data suggest that PP-007 delivery primes MΦs to re-establish a normal immune response to LPS challenge (i.e., comparable to what we observed in vehicle-treated WT mice) under conditions of dysregulated immunity.

In the context of a lung disease such as CF, in which hyperinflammation is coupled with bacterial infection, anti-inflammatory treatments may be immunosuppressive, increasing the risk of worsening infection^3^. Our data showed that PP-007 pretreatment did not weaken efficient host defense, as measured by the initial proinflammatory cytokine production and the migration of neutrophils to the lungs in CF mice challenged with bacteria (Fig. 5d–f). However, pretreatment was sufficient to accelerate the resolution of lung neutrophilia in CF mice, decrease the numbers of Ly6C^+^ inflammatory monocytes and activated T cells, and normalize the levels of the proinflammatory cytokines IL-6, IL-17, IL-12p70, and IP-10 in BALF samples (Fig. 5h). We hypothesize that the reduced number of neutrophils in PP-007-treated CF mice could, among other factors, be ascribed to the profound effect of the drug on the levels of IL-17, given the importance of this cytokine in the persistence of lung neutrophilia. Importantly, PP-007 pretreatment did not weaken the host defense against *PA* in a model of chronic bronchial bacterial infection (Supplementary Fig. 7).

One strength of this work is the reproducibility of PP-007 effects under different experimental conditions and in animal models of lung hyperinflammation. For example, the anti-inflammatory properties of PP-007 were recapitulated in a βENaC-Tg mouse model featuring airway mucus obstruction, a characteristic of CF and other muco-obstructive lung diseases (Supplementary Fig. 5). PP-007 may have additional benefits compared with inhaled CO, especially in diseases, such as CF^48,49^, in which systemic inflammation is a concern or thick mucus may prevent the full penetration of an inhaled medication into the lower airways. In addition, PP-007 also acts on hypoxic tissues and protects against oxidant-induced damage, an additional benefit for diseases characterized by hypoxia, such as CF and muco-obstructive pulmonary disease.

**Fig. 7:**
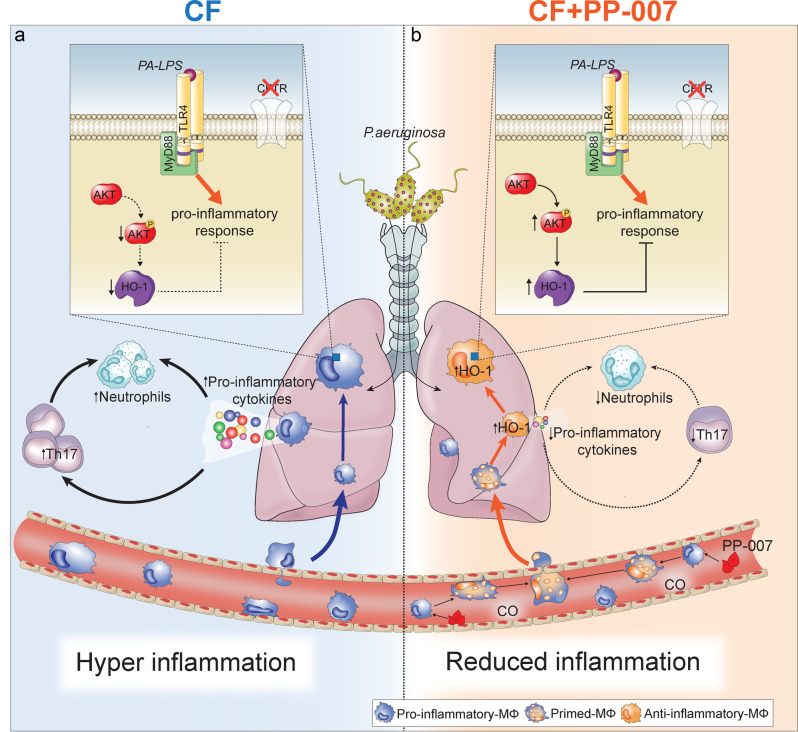
Proposed mechanism of action of PP-007 involved in resolving lung inflammation in CF. **a** During infection, circulating monocytes migrate to the lungs. Because of the defective induction of HO-1, recruited CF MΦs fail to acquire an anti-inflammatory phenotype. This leads to an enhanced nonresolving proinflammatory environment with elevated numbers of IL-17-producing cells that may contribute to lung neutrophilia. **b** PP-007 induces high levels of HO-1 in circulating monocytes recruited to lung tissue. Rescue of HO-1 expression primes lung monocyte-derived MΦs to acquire an anti-inflammatory profile that helps to reduce proinflammatory cytokine levels and IL-17-producing cell recruitment and to expedite the elimination of lung neutrophils.

In summary, our work elucidates the mechanism of action by which PP-007 improves the resolution of inflammation (summarized in Fig. 7). Moreover, we demonstrate the potential utility of PP-007 in controlling neutrophilic pulmonary inflammation in the context of diseases such as CF and muco-obstructive lung diseases.

## Supplementary information

Supplementary Informations
