# Supplementary material for: Recruitment of monocytes primed to express heme oxygenase-1 ameliorates pathological lung inflammation in cystic fibrosis
Source: Exp Mol Med. 2022 May 17;54(5):639–52. doi: 10.1038/s12276-022-00770-8 (PMC9166813; doi:10.1038/s12276-022-00770-8)

Title

Recruitment of monocytes primed to express heme oxygenase-1 ameliorates pathological lung inflammation in cystic fibrosis.

Short title: HO-1 expressing monocytes treat lung hyper-inflammation

Authors

Caterina Di Pietro[1], Hasan H Öz[1], Ping-xia Zhang[1,2], Ee-chun Cheng[1], Valentino Martis[1], Tracey L Bonfield[3], Thomas J Kelley[3], Ronald Jubin[4], Abraham Abuchowski[4], Diane S Krause[2], Marie E Egan[1,5], Thomas S Murray[1] and Emanuela M Bruscia[1 *]

Affiliations

[1]Departments of Pediatrics, Yale University School of Medicine, New Haven CT, USA

[2]Laboratory Medicine and the Yale Stem Cell Center, Yale University School of Medicine, New Haven CT, USA

[3]Department of Genetics and Genome Sciences, Case Western Reserve University, Cleveland, Ohio;

[4]Prolong Pharmaceuticals, South Plainfield, NJ, USA.

[5]Cellular and Molecular Physiology, Yale University School of Medicine, New Haven CT, USA;

*Corresponding author. Email: emanuela.bruscia@yale.edu

Competing interests:

The authors declare the following competing financial interests: the Bruscia lab has been supported by a grant from Prolong Pharmaceuticals.

Supplementary Figures:

1. related to Figure 2: PP-007 induces HO-1 in macrophages via the synergistic activation of the MyD88 and PI3K/AKT pathways.

2. related to Figure 3: PP-007 allows a normal induction of HO-1 in CF MΦs.

3. related to Figure 4: Systemic delivery of PP-007 induces HO-1 expression in lung macrophages.

4. related to Figure 5: Systemic delivery of PP-007 reduces the inflammatory response in CF lungs.

5. related to Figure 5: Systemic delivery of PP-007 reduces inflammation in βENaC-Tg mice.

6. Related to Figure 5: Systemic delivery of PP-007 does not affect the inflammatory response in WT mice.

7. Related to Figure 5: PP-007 does not weaken the host defense against *P. aeruginosa.*

Supplementary Table 1: Genotypes of CF patients enrolled in this study.

Supplementary Table 2: List of mice used for in vivo LPS treatment.

Supplementary Fig. 1 related to Fig. 2: PP-007 induces HO-1 in macrophages via the synergistic activation of the MyD88 and PI3K/AKT pathways.

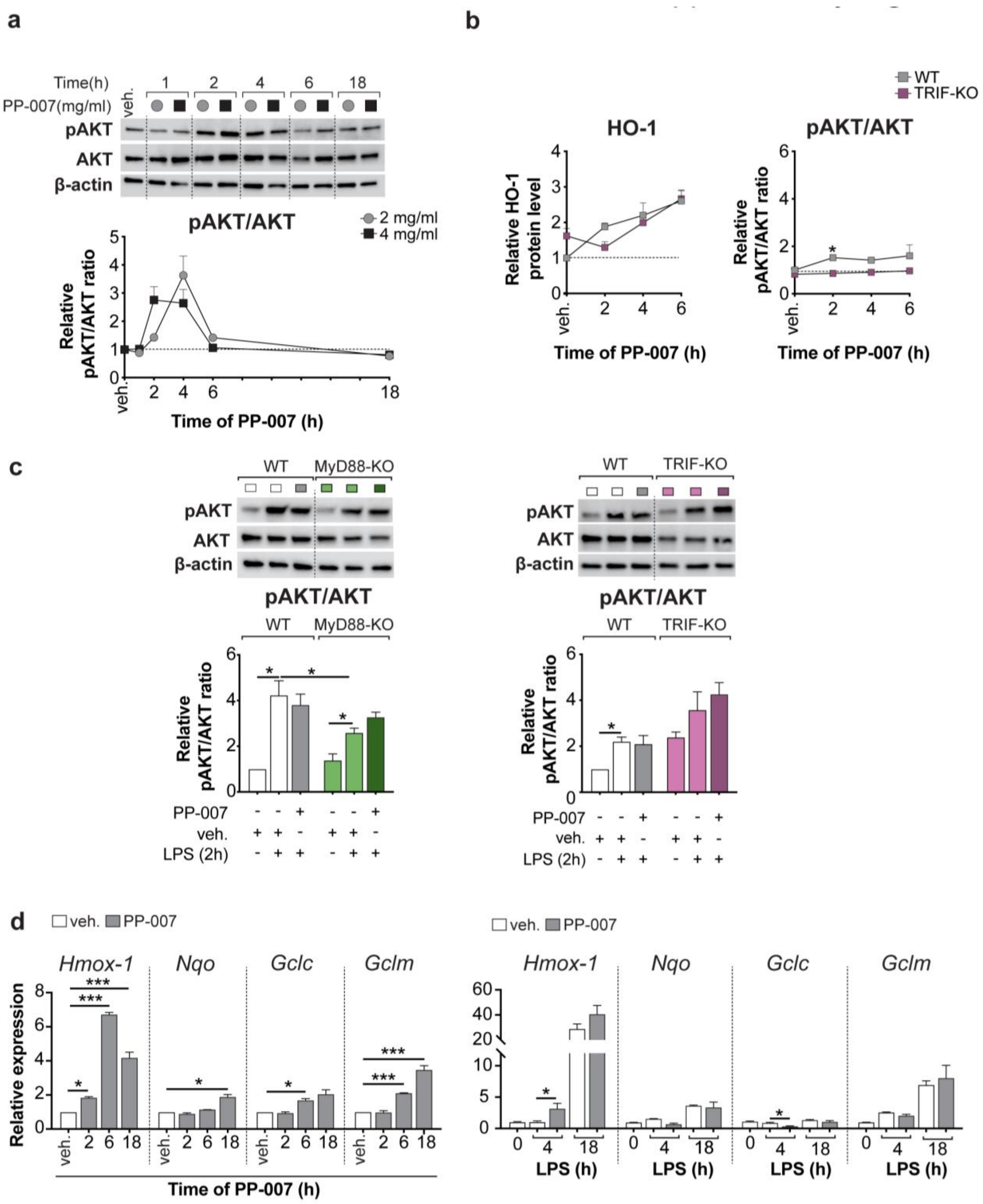

(**a**) Representative WB and densitometric analysis for pAKT and AKT in WT MΦs treated with 2 mg/ml or 4 mg/ml of PP-007 for the time indicated. Data are represented as fold increase of PP-007-treated samples relative to vehicle-treated samples (Time 0) and are the results of two biological repeats. (**b**) Densitometric analysis for HO-1, pAKT, and AKT in WT and TRIF-KO MΦs treated with 2 mg/ml of PP-007 for the time indicated. Time 0 corresponds to the samples treated with vehicle. (**c)** Representative WB and densitometric analysis of pAKT and total AKT in WT and MyD88-KO (left panel) or TRIF-KO (right panel) pre-treated with vehicle or 2 mg/ml of PP-007 for 6 h, then challenged with PA-LPS for an additional 2 h. (**d**) qPCR for Nrf2-target genes (*Hmox-1*, *Nqo1, Gclc* and *Gclm*) in WT MΦs treated with 2 mg/ml of PP-007 for the time indicated (left) and following additional PA-LPS stimulation for 4h and 18 h started after 6 h of PP-007 pretreatment (right). Time 0 corresponds to the sample treated with vehicle and not treated with LPS. mRNA levels are normalized to 18S. For WB, band intensity is normalized for the corresponding β-Actin intensity, and the rate of AKT phosphorylation is shown as the ratio of phosphoprotein to total protein. Data are represented as fold increase relative to WT vehicle-treated samples. Data are represented as means ± SEM and, unless otherwise indicated, are the result of three biological repeats. Statistical analyses were conducted using a two-tailed unpaired Student's t test with unequal variance: *$P \leq 0.05$, **$P < 0.01$, and ***$P < 0.001$. In (**b**), * symbols indicate a statistically significant difference between WT and TRIF-KO samples. Cropped blots are displayed, and full-length gels and blots are included in the **Supplementary Material**.

Supplementary Fig. 2 related to Fig. 3: PP-007 allows a normal induction of HO-1 in CF MΦs.

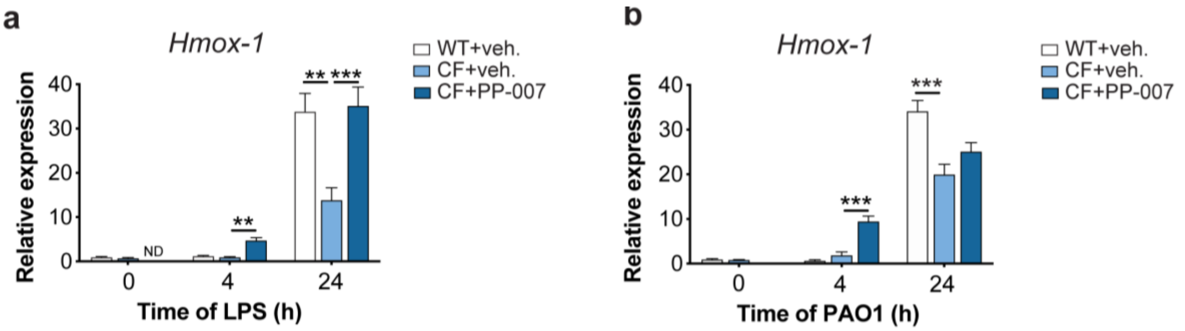

(**a**) qPCR for *Hmox-1* in CF MΦs pre-treated with vehicle or 2 mg/ml of PP-007 for 6 h, before the addition of PA-LPS or (**b**) live PAO1 for an additional 4 h and 18 h. WT MΦs pre-treated with vehicle were used as control. *Hmox-1* levels are normalized to 18S. Data are represented as fold increase relative to WT vehicle-treated samples without PA-LPS or PAO1. Data are represented as means ± SEM of three experimental repeats. Statistical analyses were conducted using a two-tailed unpaired Student's *t* test with unequal variance: **p < 0.01. ND: not determined

Supplementary Fig. 3 related to Fig. 4: Systemic delivery of PP-007 induces HO-1 expression in lung macrophages.

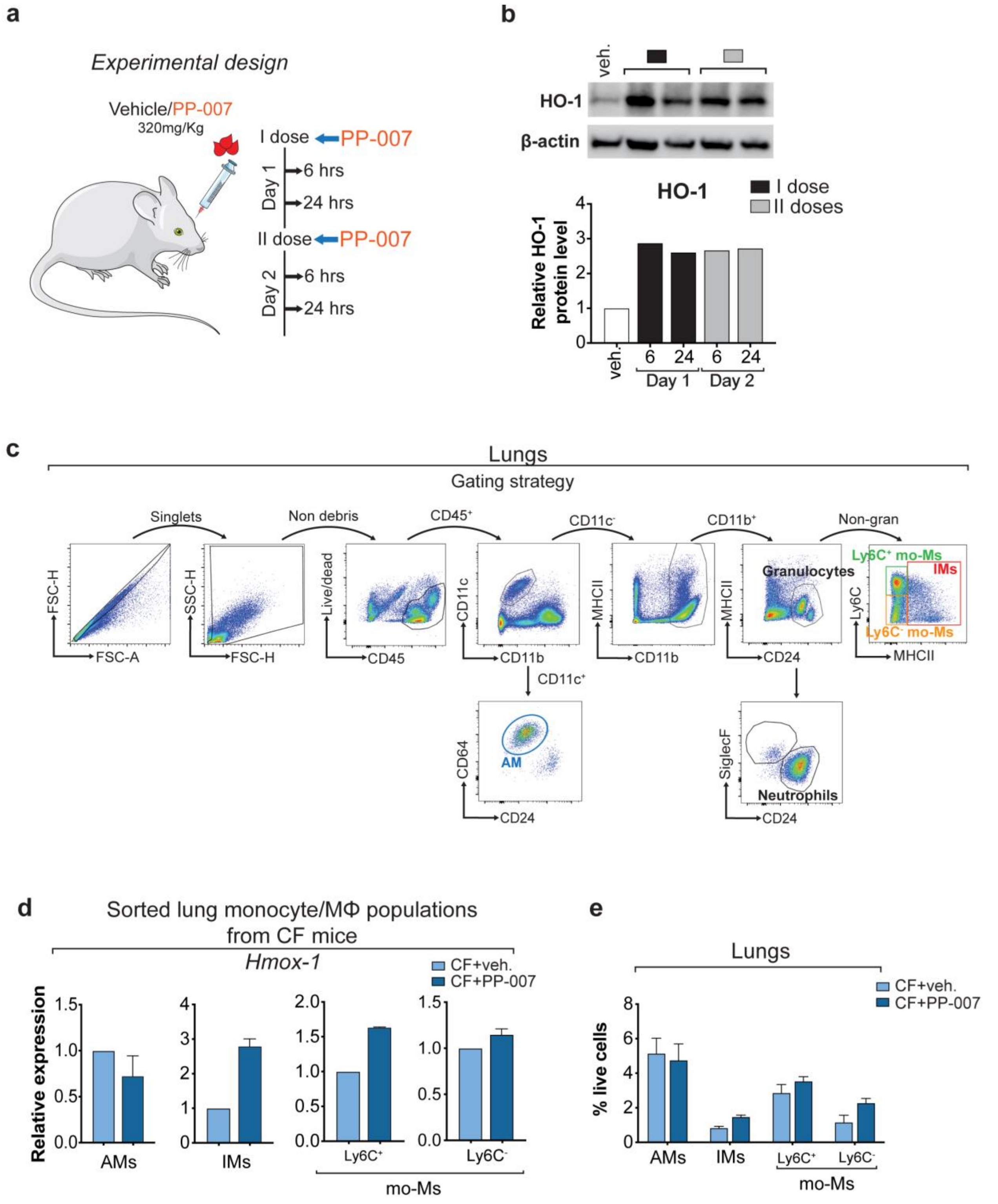

(**a**) Cartoon representing the experimental design: WT mice (1 mouse/ condition) received 1 or 2 doses of PP-007 (320 mg/kg) and were sacrificed 6 h or 24 h after each treatment. (**b**) Representative immunoblot and densitometric analysis of HO-1 in lung lysates. Data are represented as fold increase of PP-007-treated sample relative to vehicle-treated sample. (**c**) Sequential gating strategy used to identify monocyte/macrophages

population and neutrophils in the lung. After the exclusion of doublets and debris, cell populations were gated from viable CD45$^+$ cells: alveolar macrophages (AMs): CD11c$^+$CD64$^+$; neutrophils: CD11c$^-$CD11b$^+$CD24$^+$Ly6G$^+$; after exclusion of granulocytic cells: interstitial macrophages (IMs): CD11c$^-$CD11b$^+$MHC-II$^+$; and monocyte-derived macrophages (mo-Ms): CD11c$^-$CD11b$^+$MHC-II$^-$Ly6C$^+$ and CD11c$^-$CD11b$^+$MHC-II$^-$Ly6C$^-$. (**d**) qPCR for *Hmox-1* from monocyte/macrophages population sorted from lung of CF mice 24 h after PP-007 or vehicle treatment (n=2/group). (**e**) Percentage of monocyte/macrophages out of live cells in the lung of CF mice 24 h after PP-007 or vehicle treatment (n=3/group).

Supplementary Fig. 4 related to Fig. 5: Systemic delivery of PP-007 reduces the inflammatory response in CF lungs.

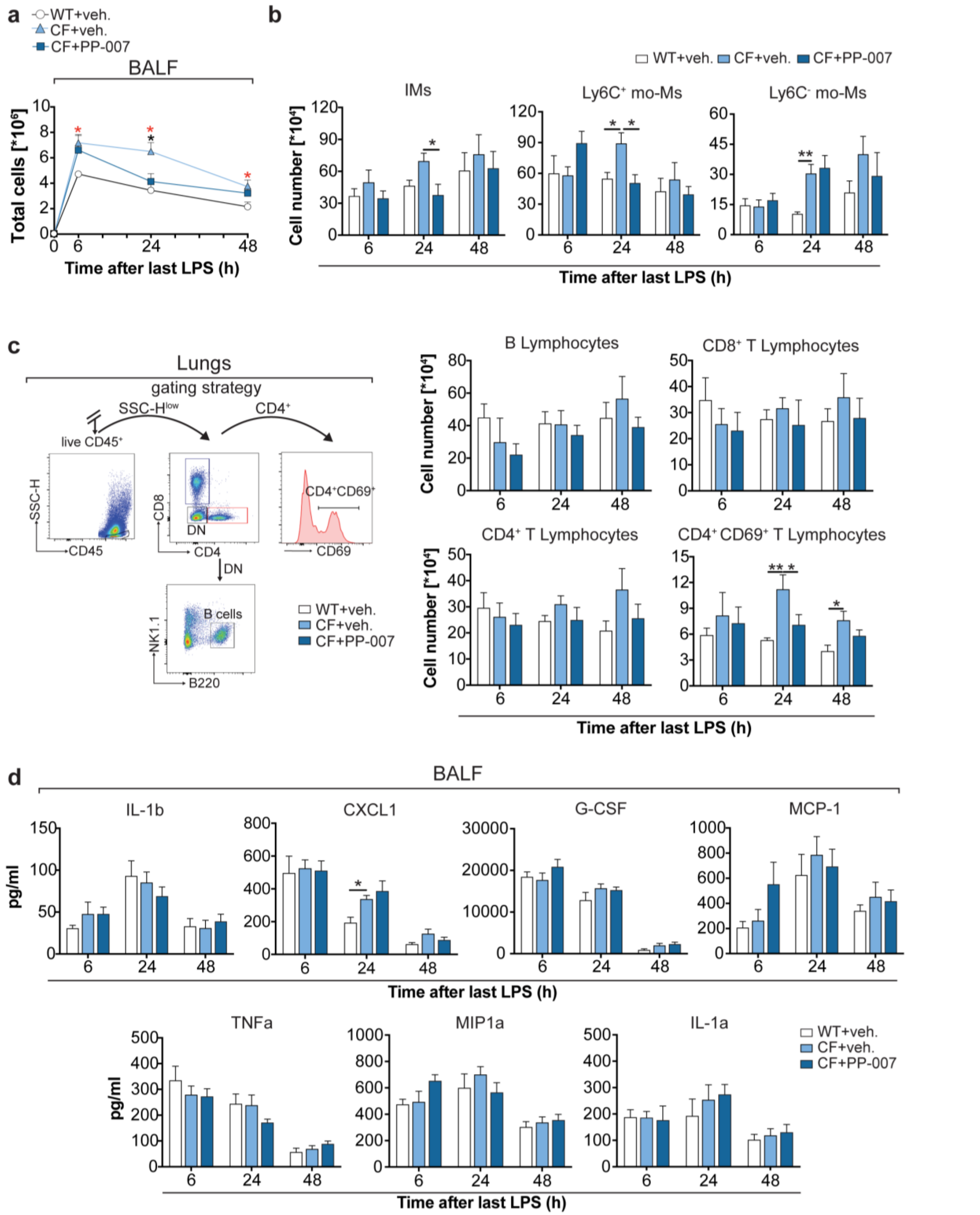

(**a**) Total cell counting in the bronchoalveolar lavage (BAL) fluid of WT and CF mice treated with vehicle and CF mice treated with PP-007 at the time indicated. (**b**) Number of monocyte/macrophages in the lung tissues.

(**c**) Gating strategy (left) and number of B and T lymphocytes in the lung (right). After the exclusion of doublets and debris, cell populations were gated from viable $CD45^+$ cells. T lymphocytes were distinguished based on CD4 and CD8 markers, and CD4 cells were further analyzed for the expression of the activation marker CD69. B lymphocytes were gated as $B220^+$ from DN population ($CD4^-CD8^-$). For **b** and **c** the percentage of cells in the live/singlets gate was then multiplied by the number of live cells to obtain an absolute live-cell count. AMs: alveolar macrophages; IMs: Interstitial macrophages; mo-Ms: monocyte-derived macrophages. (**d**) Cytokine concentration in BALF. Graphs show means ± SEM. In **a** Red symbol (*) indicates a statistically significant difference between vehicle-treated WT and CF groups. Black symbol (*) indicates a statistically significant difference between vehicle-treated CF group and PP-007-treated CF group. A detailed list of mice used for each experiment is included in **Supplementary Table 2**. Statistical analyses were conducted using a two-tailed unpaired Student's *t* test with unequal variance: $*P \leq 0.05$ and $**P < 0.01$.

Supplementary Fig. 5 related to Fig. 5: Systemic delivery of PP-007 reduces inflammation in βENaC-Tg mice.

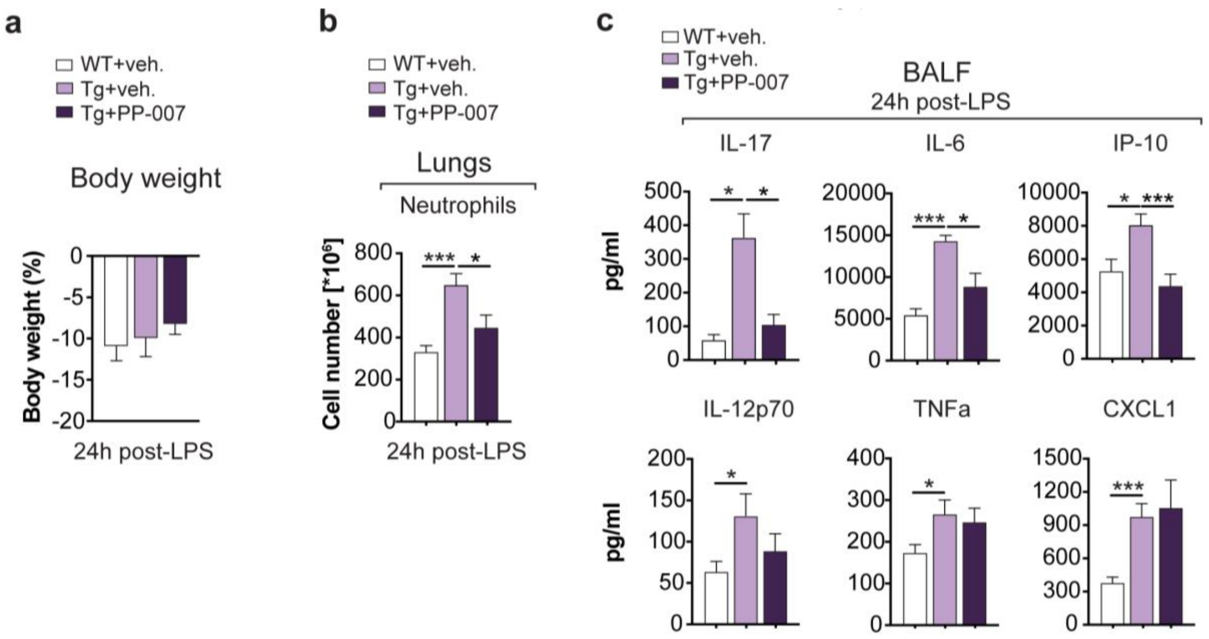

βENaC-Tg (Tg) mice were pre-treated with vehicle (n=7) or PP-007 (n=10), then nebulized with PA-LPS as depicted in **Fig. 5a**. βENaC-Tg WT mice (WT; n=9-11 mice) were pre-treated with vehicle and used as control. Mice were analyzed 24 h after the last LPS nebulization. (**a**) Weight loss as percentage of body weight. (**b**) Number of neutrophils in lung parenchyma assessed by flow cytometry. (**c**) Cytokine concentration in BALF. Graphs show means ± SEM and are a combination of three independent experiments. Statistical analyses were conducted using a two-tailed unpaired Student's *t* test with unequal variance: *$p < 0.05$, **$p < 0.01$, and ***$p < 0.001$.

Supplementary Fig. 6 Related to Fig. 5: Systemic delivery of PP-007 does not affect the inflammatory response in WT mice.

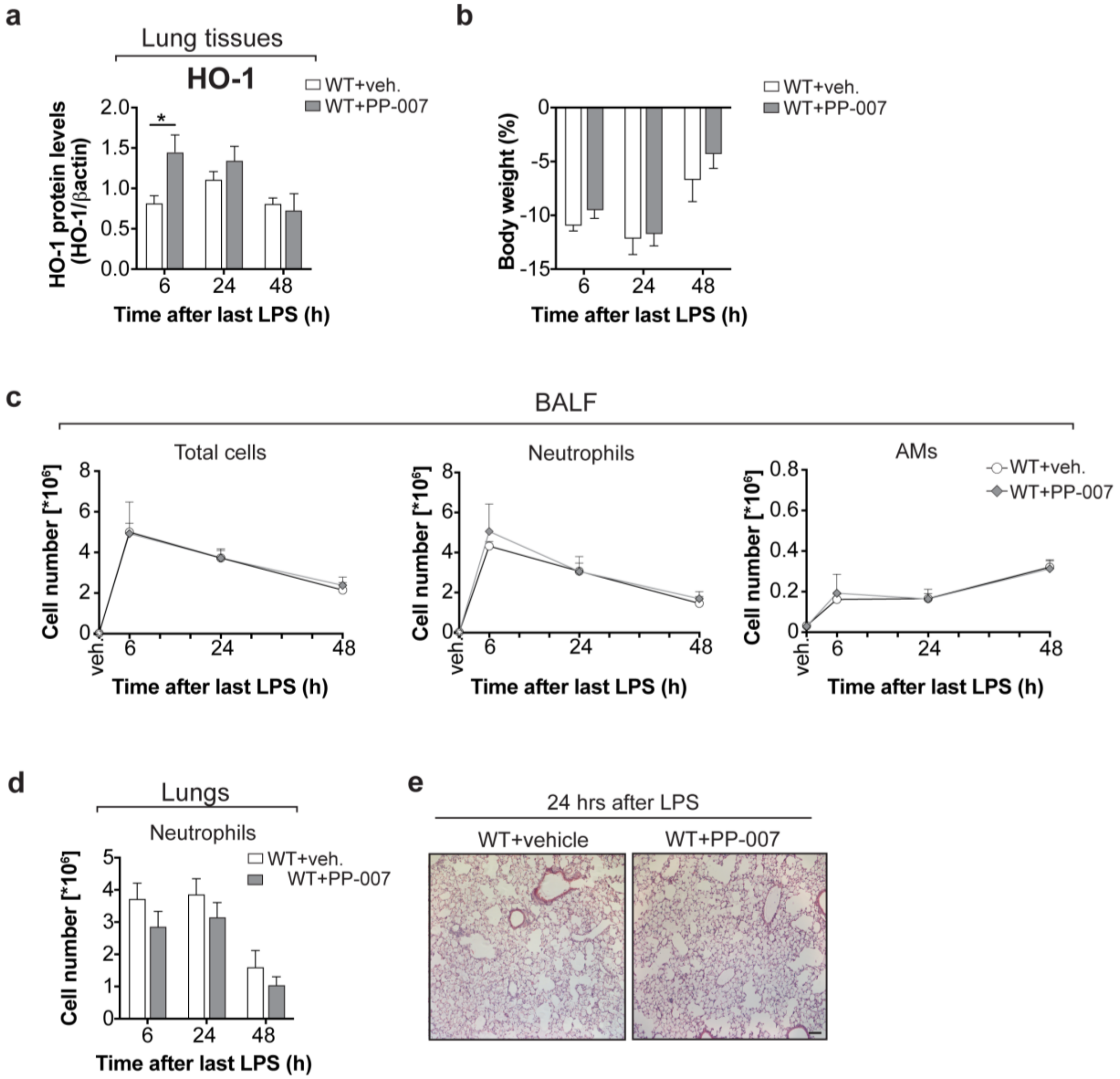

WT mice were pre-treated with vehicle or PP-007, then nebulized with PA-LPS as depicted in Fig. 5a. (**a**) Densitometric analysis of HO-1 in lung lysates. Intensities of HO-1 immunoreactive signals were measured and normalized to β-Actin. (**b**) Weight loss as percentage of body weight. (**c**) Total and differential cell counting in the BALF. (**d**) Number of neutrophils in lung parenchyma assessed by flow cytometry using sequential gating strategy shown in **Supplementary Fig. 3**. The percentage of cells in the live/singlets gate was then multiplied by the number of live cells to obtain an absolute live-cell count. (**e**) Representative hematoxylin/eosin staining in paraffin-embedded lung tissues at 24 h from the last LPS. Magnification: x10; Scale bar: 100 µm. Graphs show means ± SEM. A detailed list of mice used for each experiment is included in **Supplementary Table 2**. Statistical analyses were conducted using a two-tailed unpaired Student's *t* test with unequal variance: **P* < 0.05.

Supplementary Fig. 7 Related to Fig. 5: PP-007 does not weaken the host defense against *P. aeruginosa.*

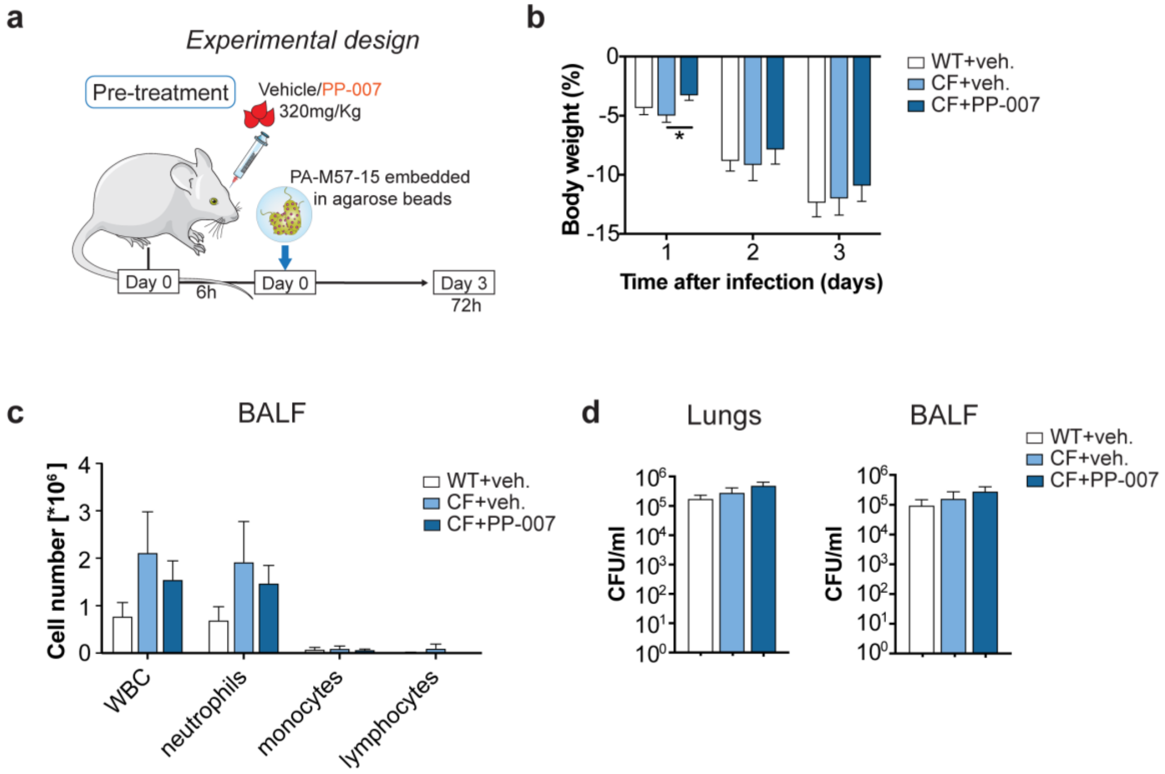

(**a**) Schematic cartoon showing the treatment: CF mice were injected I.V. with 1 dose of PP-007 (320 mg/kg) (n=10) or vehicle (n=6), and WT mice (n=6) were injected with vehicle and used as control. At 6 h after injection, PA-M57-15 was instilled into the right mainstem bronchus at a sub-lethal dose of 10^5 viable cfu/ml embedded in agarose beads. Mice were sacrificed 3 days post-infection (72 h). (**b**) Weight loss as percentage of body weight. (**c**) Total and differential cell counting in the BALF. (**d**) PA CFUs from the lung and BALF. Statistical analyses were conducted using a two-tailed unpaired Student's *t* test with unequal variance: *$p < 0.05$

Supplementary Table 1: Genotypes of CF patients enrolled in this study.

Patient ID	Mutation
1	F508del/unknown
2	F508del/F508del
3	F508del/F508del
4	F508del/F508del
5	F508del/M470V
6	F508del/unknown
7	F508del/F508del
8	F508del/W846X
9	F508del/F508del
10	F508del/F508del
11	F508del/F508del
12	F508del/F508del
13	F508del/F508del
14	F508del/F508del
15	3849+10KBC>T/2215ΔG
16	G551D/M470V
17	H609R/H609R
18	1507Δ/D1270N
19	61717G>A/N1303K
20	3849+10KBC>T/2215ΔG
21	ΔF508/A613T/M470V/seq change detected: polyT 7T & 9T

Supplementary Table 2: List of mice used for in vivo LPS treatment.

Mice used for in vivo LPS treatment

	Sex	Age (wks)	Time (h)	Treat.	WB	weight	BAL cell counting	Flow	Luminex		Sex	Age (wks)	Time (h)	Treat.	WB	weight	BAL cell counting	Flow	Luminex
WT	f	20	6	Veh.					X	WT	f	16.0	6	PP-007		X			ND
WT	m	13	6	Veh.	X	X	X	X	X	WT	f	20.0	6	PP-007	X	X			ND
WT	f	13	6	Veh.	X	X	X	X	X	WT	f	24.0	6	PP-007	X	X	X	X	ND
WT	f	24	6	Veh.	X	X	X	X	X	WT	m	20	6	PP-007	X	X	X	X	ND
WT	f	24	6	Veh.	X	X	X	X	X	WT	m	24	6	PP-008	X	X	X	X	ND
WT	m	20	6	Veh.	X	X	X	X	X	WT	m	22	6	PP-009	X	X	X	X	ND
WT	f	20	6	Veh.	X	X	X	X	X										
WT	m	15	6	Veh.	X	X	X	X	X										
Number					7	7	7	7	8	Number					5	6	4	4	
WT	f	16	24	Veh.	X					WT	f	16	24	PP-007	X		X		ND
WT	m	25	24	Veh.	X					WT	m	28	24	PP-007	X	X	X		ND
WT	f	12	24	Veh.	X					WT	m	20	24	PP-007	X	X	X	X	ND
WT	m	12	24	Veh.	X	X				WT	f	20	24	PP-007	X	X	X	X	ND
WT	f	24	24	Veh.	X	X				WT	f	20	24	PP-007	X	X	X	X	ND
WT	f	24	24	Veh.	X	X		X	X	WT	f	16	24	PP-007	X	X	X	X	ND
WT	f	20	24	Veh.	X	X		X	X	WT	f	16	24	PP-007	X	X	X	X	ND
WT	f	16	24	Veh.	X	X		X	X	WT	f	12.0	24	PP-008			X	X	ND
WT	f	16	24	Veh.	X	X	X	X	X	WT	m	12.0	24	PP-009			X	X	ND
WT	f	16	24	Veh.	X	X	X	X	X										
WT	f	16	24	Veh.	X	X	X	X	X										
WT	f	16	24	Veh.	X	X	X	X	X										
WT	f	16	24	Veh.	X	X	X	X	X										
WT	m	25	24	Veh.	X	X	X	X	X										
WT	f	20	24	Veh.	X	X	X	X	X										
Number					15	12	7	10	9	Number					7	6	9	7	
WT	f	24	48	Veh.		X	X	X		WT	f	24	48	PP-007	X	X	X	X	ND
WT	m	24	48	Veh.	X	X	X	X	X	WT	f	24.0	48	PP-007	X	X	X	X	ND
WT	f	16	48	Veh.	X	X	X	X	X	WT	f	16	48	PP-007	X	X	X	X	ND
WT	f	14	48	Veh.	X	X	X	X	X	WT	f	15	48	PP-007	X	X	X	X	ND
WT	f	24	48	Veh.	X	X	X	X	X	WT	f	16	48	PP-007	X	X	X	X	ND
WT	m	24	48	Veh.	X	X	X	X	X	WT	f	10.0	48	PP-007	X	X	X	X	ND
WT	f	12	48	Veh.	X	X	X	X	X										
WT	f	12	48	Veh.	X	X	X	X	X										
Number					7	8	8	8	7	Number					6	6	6	6	
										CF	m	26	6	PP-007		X			
										CF	f	20	6	PP-008		X		X	X
CF	f	25	6	Veh.	X				X	CF	m	20	6	PP-009		X		X	X
CF	f	20	6	Veh.	X	X	X	X	X	CF	f	12	6	PP-010		X		X	X
CF	f	13	6	Veh.	X	X	X	X	X	CF	f	12	6	PP-011	X	X	X	X	X
CF	f	21	6	Veh.	X	X	X	X	X	CF	m	14	6	PP-012	X	X	X	X	X
CF	f	13	6	Veh.	X	X	X	X	X	CF	f	14	6	PP-013	X	X	X	X	X
CF	m	13	6	Veh.	X	X	X	X	X	CF	f	14	6	PP-014	X	X	X	X	X
CF	m	13	6	Veh.	X	X	X	X	X	CF	f	14	6	PP-015	X	X	X	X	X
Number					7	6	6	6	7	Number					5	9	5	8	8
CF	f	25	24	Veh.	X	X				CF	f	20	24	PP-007		X			
CF	m	16	24	Veh.	X	X				CF	f	30	24	PP-007		X			
CF	m	16	24	Veh.	X	X		X		CF	m	24	24	PP-007		X			
CF	f	20	24	Veh.	X	X		X		CF	f	24	24	PP-007		X			
CF	f	25	24	Veh.	X	X		X	X	CF	f	12	24	PP-007		X	X		
CF	f	10	24	Veh.	X	X	X	X	X	CF	f	12	24	PP-007	X	X	X		X
CF	f	16	24	Veh.	X	X	X	X	X	CF	f	12	24	PP-007	X	X	X	X	X
CF	m	20	24	Veh.	X	X	X	X	X	CF	f	12	24	PP-007	X	X	X	X	X
CF	m	20	24	Veh.	X	X	X	X	X	CF	m	16	25	PP-008	X	X	X	X	X
CF	m	12	24	Veh.	X	X	X	X	X	CF	f	16	26	PP-009	X	X	X	X	X
CF	m	12	24	Veh.	X	X	X	X	X	CF	f	20	27	PP-010	X	X	X	X	X
CF	m	12	24	Veh.	X	X	X	X	X	CF	m	16	28	PP-011	X	X	X	X	X
CF	f	16	24	Veh.	X	X	X	X	X	CF	m	20	29	PP-012	X	X	X	X	X
Number					13	13	8	11	9	Number					8	13	9	7	8
										CF	f	12	48	PP-007	X	X	X		
										CF	f	12	48	PP-007	X	X	X	X	
CF	f	12	48	Vhe.	X	X	X	X	X	CF	f	12	48	PP-007	X	X	X	X	X
CF	f	14	48	Vhe.	X	X	X	X	X	CF	f	16	48	PP-007	X	X	X	X	X
CF	f	14	48	Vhe.	X	X	X	X	X	CF	f	20	48	PP-007	X	X	X	X	X
CF	f	14	48	Vhe.	X	X	X	X	X	CF	f	12	48	PP-007	X	X	X	X	X
CF	m	14	48	Vhe.	X	X	X	X	X	CF	m	12	48	PP-007	X	X	X	X	X
Number					5	5	5	5	5	Number					7	7	5	5	5

Supplementary material: uncropped WB gels

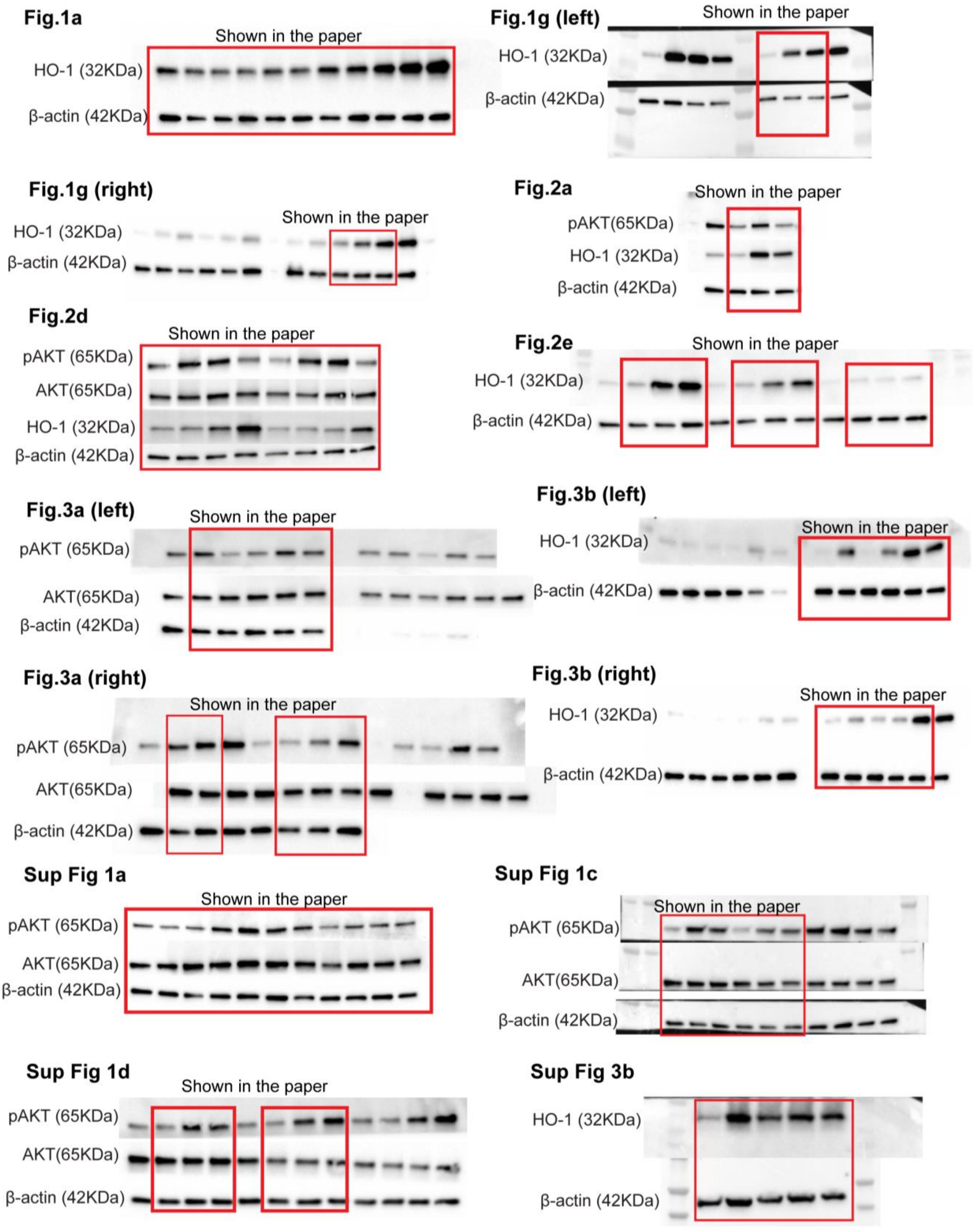

Supplement: Supplementary file 1 — Supplementary Informations [file 12276_2022_770_MOESM1_ESM.pdf]
